# GPD1L‐Mediated Glycerophospholipid Metabolism Dysfunction in Women With Diminished Ovarian Reserve: Insights From Pseudotargeted Metabolomic Analysis of Follicular Fluid

**DOI:** 10.1111/cpr.70024

**Published:** 2025-03-20

**Authors:** Jiaqi Wu, Xuehan Zhao, Ying Fang, Cong Wang, Yichang Tian, Wan Tu, Qiqian Wu, Long Yan, Xiaokui Yang

**Affiliations:** ^1^ Department of Human Reproductive Medicine, Beijing Obstetrics and Gynecology Hospital, Beijing Maternal and Child Health Care Hospital Capital Medical University Beijing China; ^2^ Key Laboratory of Organ Regeneration and Reconstruction, State Key Laboratory of Stem Cell and Reproductive Biology Institute of Zoology, Chinese Academy of Sciences Beijing China; ^3^ Institute for Stem Cell and Regeneration Chinese Academy of Sciences Beijing China; ^4^ University of Chinese Academy of Sciences Beijing China; ^5^ Beijing Institute for Stem Cell and Regenerative Medicine Beijing China

**Keywords:** diminished ovarian reserve, follicular fluids, glycerophospholipid, GPD1L, metabolomics, mitochondria

## Abstract

Diminished ovarian reserve (DOR) is a pathological condition characterised by reduced ovarian function, which refers to the decreased quality and quantity of oocytes, potentially causing female infertility and various health issues. Follicular fluid (FF) serves as the microenvironment for follicular development and oocyte maturation, gaining an in‐depth understanding of the metabolic state of FF will help us uncover the key biological processes involved in ovarian aging, while the specific underlying pathogenic mechanisms are not fully understood. In this study, we utilised pseudotargeted metabolomic analysis of FF to reveal the glycerophospholipid metabolism dysfunction mediated by *GPD1L* in DOR patients. We also found that *GPD1L* was downregulated in granulosa cells (GCs) of DOR patients, resulting in increased cell apoptosis and mitochondrial dysfunction. Moreover, our results demonstrated that the downregulated expression of *GPD1L* could induce follicular atresia and impair oocyte quality in mouse ovaries. Altogether, our research suggested that *GPD1L* in GCs and the key metabolites in the glycerophospholipid metabolism pathway could potentially act as novel biomarkers of DOR diagnosis, paving the way for a new theoretical basis for understanding the pathogenesis of DOR.

## Graphical abstract

Our study demonstrated the downregulation of *GPD1L* and its mediated glycerophospholipid metabolism dysfunction in patients with diminished ovarian reserve. Moreover, we verified that *GPD1L* knockdown leads to increased cell apoptosis and mitochondrial dysfunction of KGN cells, as well as impaired oocyte quality. Our research provided a new theoretical basis for the mechanisms underlying DOR pathogenesis.

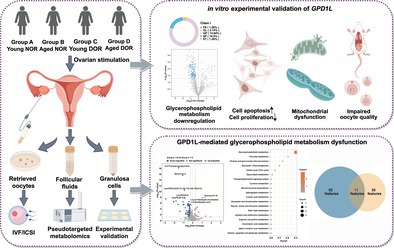

## 1 Introduction

The ovary, the crucial reproductive organ for maintaining female fertility and endocrine function, also ages earlier than most other tissues in females [1], and ovarian function highly depends on ovarian reserve. Diminished ovarian reserve (DOR) involves a reduction in both the oocyte quantity and quality, which may result in female infertility, endocrine function disorders, osteoporosis, coronary heart disease and several other health issues [2]. DOR is considered a pathological condition that is clinically characterised by indicators including decreased anti‐Müllerian hormone (AMH) level, increased follicle‐stimulating hormone (FSH) level, or a decreased antral follicle count (AFC) [3]. It was reported that the prevalence of DOR among primary infertile patients undergoing assisted reproduction has increased from 17.2% to 23.1% in the past decade [4]. The aetiology of DOR is complex, involving environmental, genetic, immune, iatrogenic factors and other aspects, while most patients have idiopathic DOR with unknown causes [5, 6, 7]. Therefore, it is still a great challenge in the clinical treatment of DOR due to its unclear mechanisms and limited medical therapies.

The follicular fluid (FF) is generated through the secretions of granulosa cells, theca cells, and oocytes, together with the diffusion of plasma components via the blood‐follicle barrier [8, 9, 10]. Since the FF serves as the microenvironment for follicular growth and oocyte maturation, facilitating material and energy exchange between oocytes and surrounding cells, mainly granulosa cells, the changes in its components may represent the metabolic status and function of oocytes and granulosa cells [11]. It is reported that the alternation of metabolites in FF impacts oocyte maturation, follicular wall rupture, sperm‐mediated oocyte activation, and embryo development, even on female reproductive function [12, 13]. Thus, in‐depth studies on the metabolic profiles of FF may provide a new perspective and enhance the comprehension of the mechanisms of ovarian aging, particularly DOR.

Metabolomics can qualitatively and quantitatively detect metabolic alterations, which can reflect the biological events downstream of gene expression in physiological and pathological changes [14]. In recent years, metabolomics has also been used to study the metabolic status of FF, which may contribute to revealing the pathogenic mechanism and identifying potential biomarkers. It was reported that the lipid metabolisms of FF changed in females at different ages, which may be related to the quality of oocytes [15]. Targeted metabolomics analysis revealed reduced polyunsaturated choline plasmalogen levels and a decreased dimethylarginine/arginine ratio in the FF of women with DOR [16]. Another research identified significantly changed oxylipin metabolite levels in FF, especially arachidonic acid metabolism, resulting in decreased fertility in DOR patients [17]. Though various metabolomics analyses have revealed that metabolite alterations are associated with DOR, their specific roles in granulosa cell dysfunction and follicular atresia still need to be explored.

Glycerophospholipids are the most abundant lipids of mammalian cell membranes, a class of amphiphilic lipids consisting of a glycerol backbone attached to phosphate head groups and hydrophobic fatty acyl chains [18], which are critical to the integrity and stability of cellular and subcellular structures and functions [19]. Impaired glycerophospholipid metabolism has been reported to be related to several disorders, including respiratory diseases [20], diabetes mellitus [21], autoimmune diseases [22], neurological diseases [18] and cancers [23]. The glycerophospholipid metabolism pathway is regulated by multiple genes, among which glycerol‐3‐phosphate dehydrogenase 1‐like (*GPD1L*) encodes the crucial enzyme (glycerol‐3‐phosphate dehydrogenase) GPDH [24]. *GPD1L* has been previously studied in hereditary arrhythmias and cancer progression [25, 26, 27], while its association with ovarian aging has been less explored.

In this study, we performed a pseudotargeted metabolomic analysis of FF samples from 120 women undergoing in vitro fertilisation and embryo transfer (IVF‐ET) utilising ultra‐high‐performance liquid chromatography‐triple‐quadrupole mass spectrometry (UHPLC‐TQMS), revealing downregulation of the glycerophospholipid metabolism pathway in patients with DOR and advanced age. Moreover, we identified a reduced expression of *GPD1L*, the gene that encodes the key enzyme of this pathway in granulosa cells of both DOR and aged patients. Furthermore, by in vitro experiments, we validated that *GPD1L* down‐expression could induce cell apoptosis, lead to abnormal mitochondrial morphology and impaired mitochondrial function, as well as induce follicular atresia and decrease oocyte quality. Altogether, our findings demonstrated the *GPD1L*‐mediated glycerophospholipid metabolism dysfunction in patients with DOR, offering new insights into the mechanisms of ovarian aging.

## 2 Results

### 2.1 The Metabolic Profiles of Follicular Fluids Are Modified in Patients With DOR and Advanced Age

**FIGURE 1:**
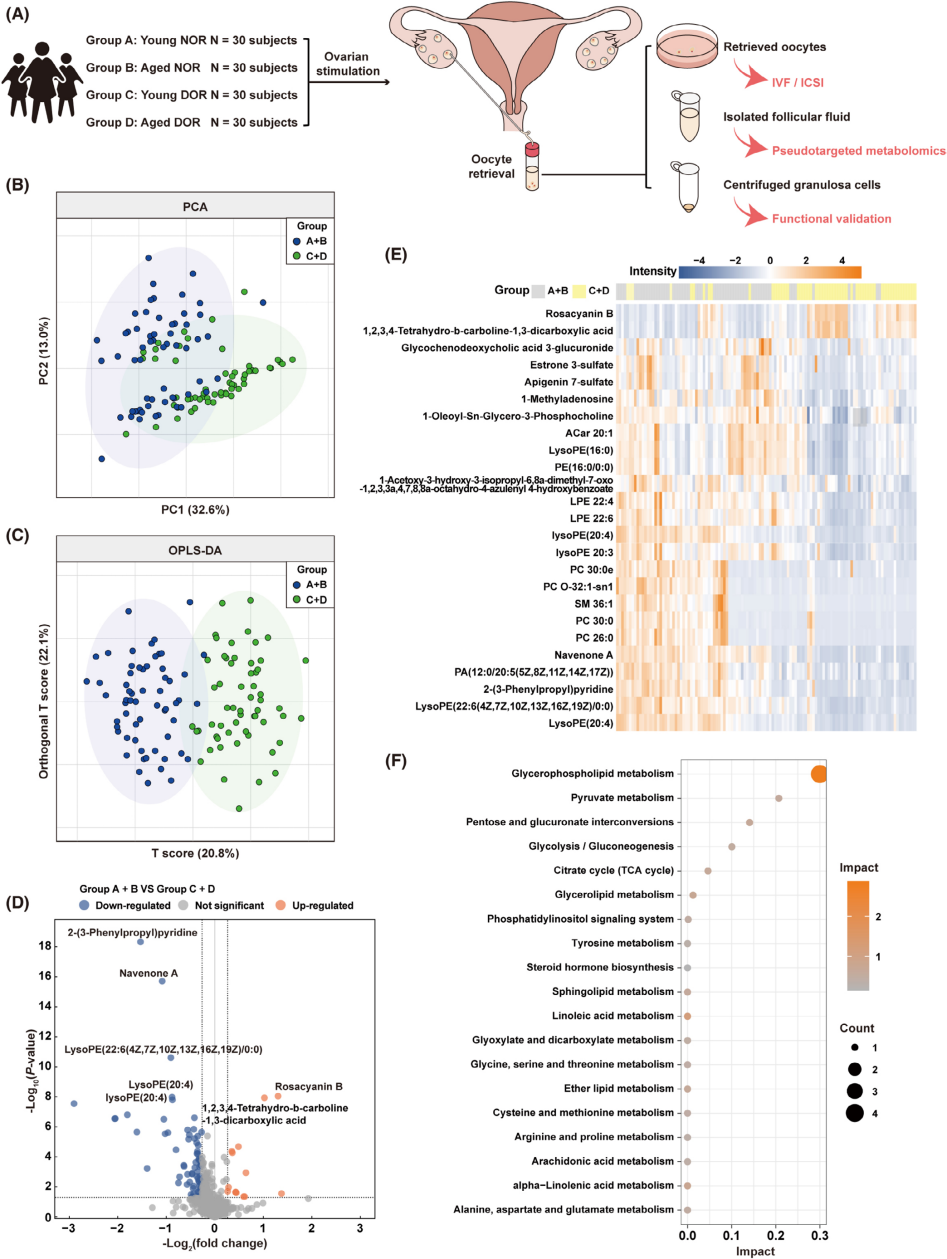
The metabolic profiles of follicular fluids between NOR and DOR groups. (A) Schematic diagram of human follicular fluids collection. (B) The PCA scores scatter plot comparing follicular fluid samples from NOR and DOR groups. (C) The OPLS‐DA scores scatter plot comparing follicular fluid samples from NOR and DOR groups. (D) Volcano plot showing differential metabolites between NOR and DOR groups. Orange dots for upregulation and blue dots for downregulation in the DOR group. (E) Heatmap showing the levels of the representative differential metabolites in NOR and DOR groups. The colour bar indicates low to high levels of metabolisms from blue to orange. (F) Bubble plot showing the representative KEGG pathways enriched with the differential metabolites. The size of the bubble represents the amounts of metabolites enriched in the pathways.

To obtain a direct metabolic profile of FF in patients with DOR, we performed a pseudotargeted metabolomic analysis of FF from 120 women undergoing IVF‐ET, including 60 DOR patients and 60 patients with normal ovarian reserve (NOR). Each group was then divided into two subgroups (30 patients for young and 30 for aged groups) (Figure 1A). The demographic and clinical profiles of the studied population are shown in Table S1. After validating the data type, distribution of MS intensity and correlation analysis based on quality control samples (QCs) (Figure S1A–C), metabolome data from 119 patients were retained for further statistical analyses and 608 metabolites were identified in all FF samples. Firstly, the separation of metabolic profiles was assessed by principal component analysis (PCA) and orthogonal partial least squares discriminant analysis (OPLS‐DA) in which PCA initially showed a clustering trend between the NOR and DOR groups (Figure 1B), while OPLS‐DA further demonstrated more distinct discriminations between the two groups, suggesting a significant alternation in metabolites (Figure 1C). Additionally, the young and aged groups also showed differences in metabolite features in FF samples based on PCA and OPLS‐DA (Figure S2A,B). Next, the differential metabolites were further identified by the Mann‐Whitney U test, fold change, and variable importance in the projection (VIP) values. Metabolites with *p* < 0.05, |fold change (FC)| > 1.2 and VIP > 1 were considered significant. Consequently, 65 differential metabolic features were identified between the NOR and DOR groups, whereas 48 differential metabolic features were noted between the young and aged groups (Figure 1D, Figure S2C). The top 25 differential metabolites in each comparative group were shown in the heatmaps (Figure 1E, Figure S2D). Furthermore, enrichment analyses were performed using the Kyoto Encyclopedia of Genes and Genomes (KEGG) database to identify the pathways associated with the differential metabolites. KEGG analysis showed a variety of altered metabolic pathways, with glycerophospholipid metabolism being the most enriched (Figure 1F, Figure S2E). Taken together, we have demonstrated the altered metabolic profile of FF in patients with DOR and patients with advanced age, among which the glycerophospholipid metabolism pathway is the most affected.

### 2.2 The Glycerophospholipid Metabolism Pathway Is Downregulated in Patients With DOR and Advanced Age

**FIGURE 2:**
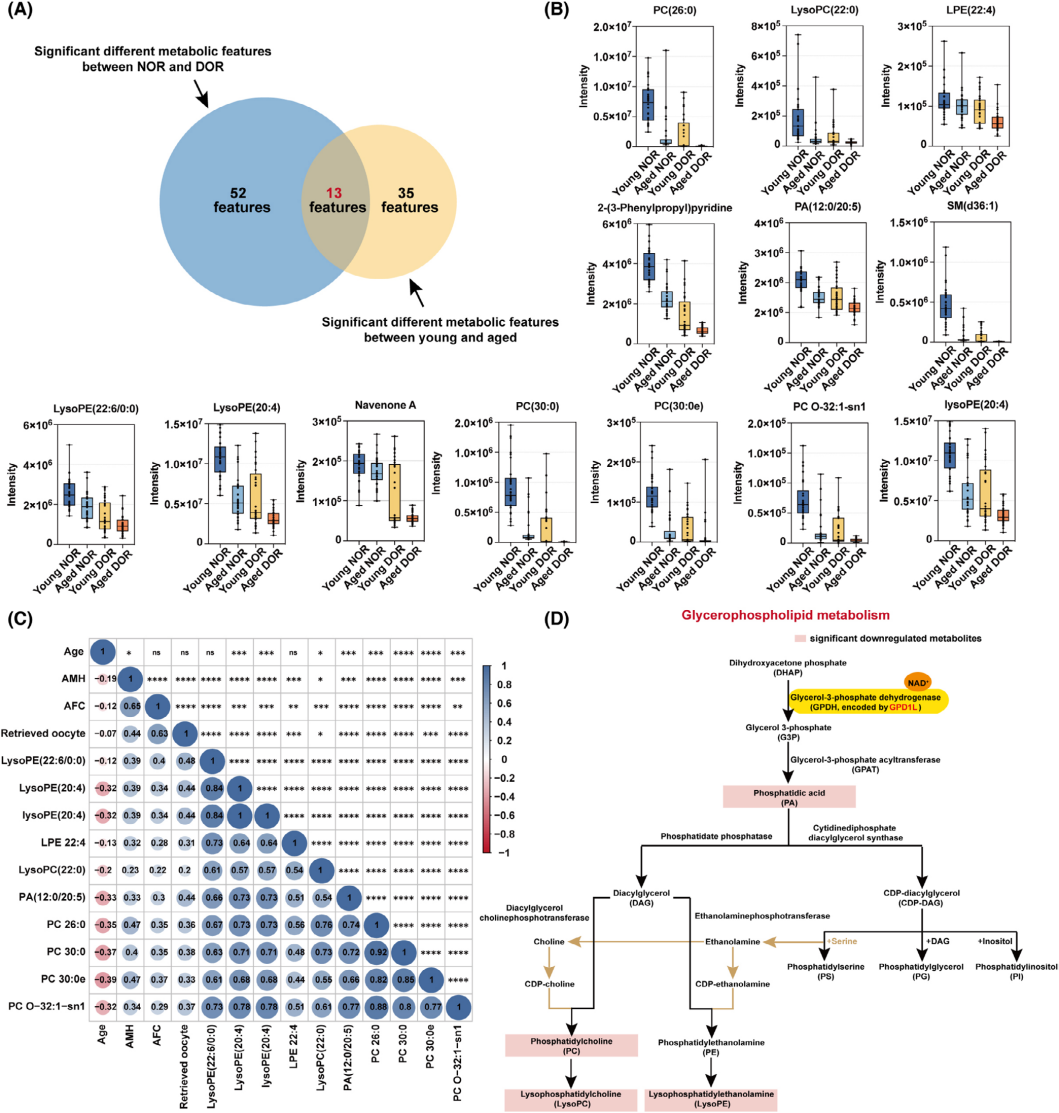
The glycerophospholipid metabolism pathway is downregulated in patients with DOR and advanced age. (A) Diagram showing the same differential metabolic features identified in both two comparative groups. (B) Comparisons of the levels of the same differential metabolic features in follicular fluids among four groups. (C) Pearson correlation coefficients for ovarian function indicators (female age, AMH, AFC, and retrieved oocyte). The dots' size represents the magnitude of the correlation. The colour bar from blue to red indicates the correlation from positive to negative. (D) Diagram illustrating the glycerophospholipid metabolism pathway and the down‐regulated key metabolic features in the pathway. *p < 0.05, **p < 0.01, ***p < 0.001, ****p < 0.0001, ns, not significant.

Given our observation of altered metabolic profiles in both DOR patients and aged patients, we subsequently analysed whether there were any metabolites in their FF that were co‐altered. The results showed that among all the differential metabolic features, 13 features coexisted between two different comparative groups (Figure 2A). Next, we detected the levels of these 13 metabolites in four subgroups respectively and found that all the metabolites showed a significant decline along with the progression of DOR or the increase in age (Figure 2B).

To further explore the association between the levels of these metabolites and female ovarian function, indicators including age, AMH, AFC, and retrieved oocytes were analysed using Pearson correlation coefficient testing. In this context, the levels of several metabolites including phosphatidylcholine (PC), phosphatidic acid (PA), lysophosphatidylethanolamine (LysoPE) and lysophosphatidylcholine (LysoPC) exhibited a positive correlation with AMH, AFC, and retrieved oocytes number while negatively correlated with female age. Besides, the levels of these metabolites in individuals are also highly positively correlated with each other (Figure 2C). The glycerophospholipid metabolism pathway is reported as a crucial component of cell metabolism, significantly influencing cell proliferation, apoptosis, mitochondrial energy production, and various other biological processes [28]. Noticeably, 10 (LysoPE(20:4), LysoPC(22:0), LysoPE(22:6/0:0), LPE(22:4), lysoPE(20:4), PA(12:0/20:5), PC(26:0), PC O‐32:1‐sn1, PC(30:0e), PC(30:0)) of the 13 significant downregulated metabolites are crucial components of the glycerophospholipid metabolism pathway (Figure 2D), corroborating the KEGG analysis previously illustrated above. In conclusion, our results confirmed that the glycerophospholipid metabolism pathway is downregulated in DOR patients and aged patients and is highly related to female ovarian function.

### 2.3 *GPD1L* Expression Decreases in Granulosa Cells of Females With DOR and Is Highly Associated With the Decline of Ovarian Function

Considering that oocyte development and maturation are highly dependent on the metabolic supports of the surrounding somatic cells (mainly granulosa cells) and that metabolic changes in the FF can also affect the functions of granulosa cells and oocytes [9, 11], we further explored the changes related to glycerophospholipid metabolism in oocytes and granulosa cells.

**FIGURE 3:**
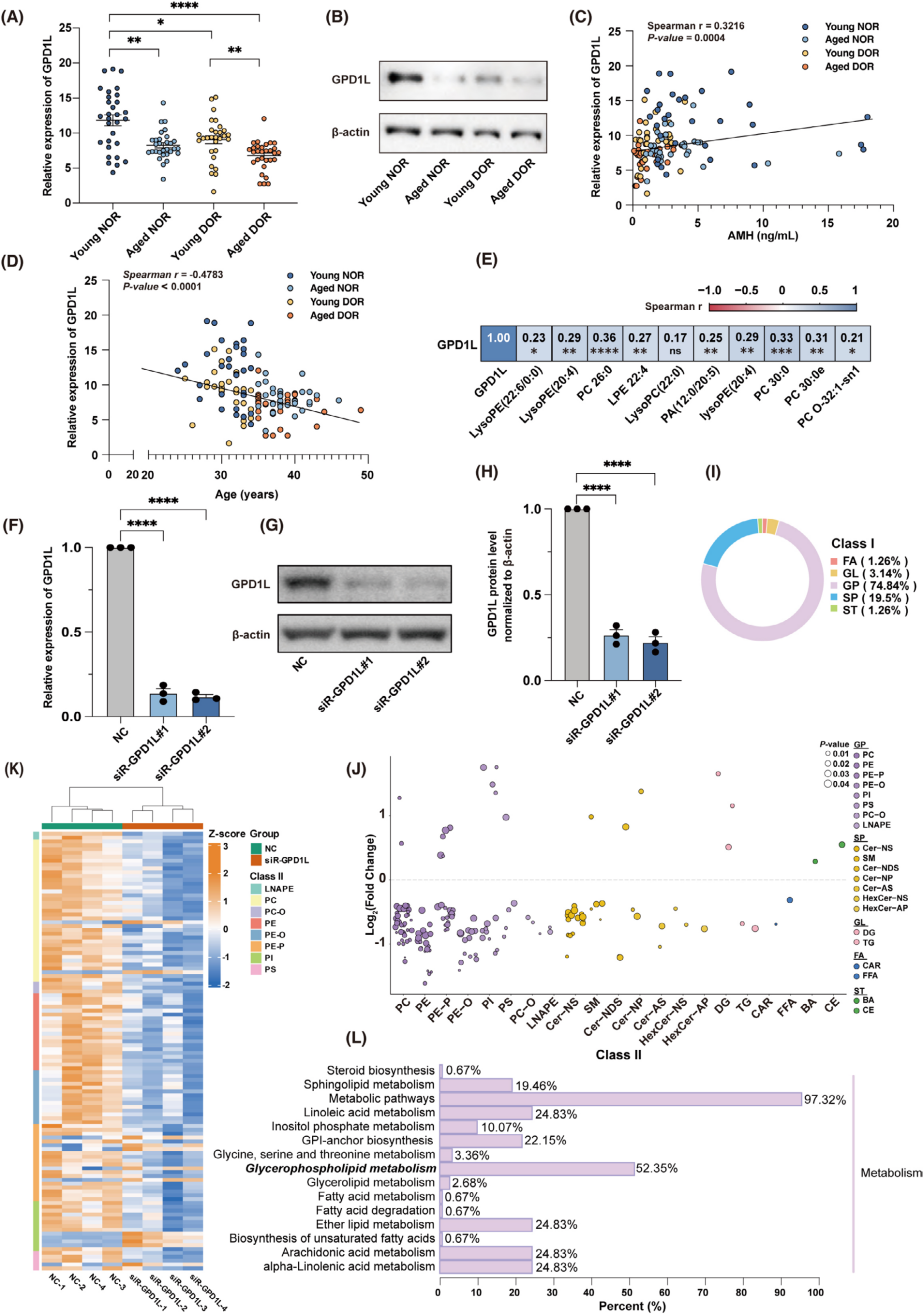
Expression levels of *GPD1L* and their correlation with ovarian function. (A) *GPD1L* expression levels in human granulosa cells from four groups detected by RT‐qPCR. (*n* = 29 for the young DOR group, *n* = 30 for the other three groups). (B) Representative western blotting images of GPD1L in human granulosa cells. (C) The correlation between *GPD1L* expression levels and AMH. (D) The correlation between *GPD1L* expression levels and female age. (E) Pearson correlation coefficients for *GPD1L* expression and metabolic features. The colour bar from blue to red indicates the correlation from positive to negative. (F) The expression levels of *GPD1L* in *GPD1L*‐KD KGN cells. (*n* = 3 independent experiments per group, gene expressions were normalised to *ACTB*). (G) Representative western blotting images of GPD1L in *GPD1L*‐KD KGN cells. (H) Quantifications of GPD1L protein levels in Figure 3G. (*n* = 3 independent experiments per group, protein levels were normalised to β‐actin). (I) Diagram showing the composition ratio of the primary classification of differential metabolites. ‐FA, fatty acyls. GL, glycerides. GP, glycerophospholipids. SP, sphingolipids. ST, sterol lipids. (J) Bubble diagram showing the variation tendency of the differential metabolites between two groups. The dots' colour represents the primary classification of metabolites. The horizontal coordinate represents the secondary classification of metabolites. Below the zero‐scale line are the downregulated metabolites and above are the upregulated metabolites in the siR‐GPD1L groups. (K) Heatmap illustrating differential metabolites levels in glycerophospholipid metabolism pathway for NC and siR‐GPD1L groups. (L) KEGG multilevel enrichment histogram showing the percent of pathways enriched with the differential metabolites in the level one classification metabolism of KEGG. All data were presented as mean ± SEM. *p < 0.05, **p < 0.01, ***p < 0.001, ****p < 0.0001, ns, not significant.

The glycerophospholipid pathway is regulated by different enzymes that are encoded by multiple genes. GPDH, as a key enzyme of the glycerophospholipid metabolism pathway, is encoded by glycerol‐3‐phosphate dehydrogenase 1 (*GPD1*) and *GPD1L* in the cytoplasm, while it is encoded by glycerol‐3‐phosphate dehydrogenase 2 (*GPD2*) in the mitochondria. Firstly, we performed bioinformatics analysis utilising the transcriptomic data from human oocytes and granulosa cells across five developmental stages based on the Gene Expression Omnibus (GEO) dataset GSE107746 [29]. The data showed the expression of *GPD1*, *GPD1L*, and *GPD2* in oocytes and granulosa cells of the five developmental stages, especially in secondary and antral stages, with higher expression levels of *GPD1L* than *GPD1*, suggesting its potentially significant role in ovarian granulosa cells (Figure S3A). Moreover, immunofluorescence (IF) staining of 3‐month‐old mice ovarian tissue confirmed that the protein levels of Gpd1l in granulosa cells of antral follicles increased compared with secondary follicles (Figure S3B). Secondly, to further assess the role of *GPD1L* in DOR, we detected the *GPD1L* mRNA levels in the granulosa cells of these patients by using RT‐qPCR. *GPD1L* was significantly decreased in DOR and aged females (Figure 3A), consistent with the decreased GPD1L protein levels shown in western blotting (WB) results (Figure 3B), further proven by the age‐associated downregulated Gpd1l levels shown in IF staining of mice ovaries (Figure S3B). In addition, considering that oxidative stress is widely reported as a key factor contributing to ovarian aging [30, 31, 32], we supplemented 50 μm hydrogen peroxide (H_2_O_2_) into the culture medium of human granulosa‐like cells (KGN cells) for 7 days to simulate the chronic oxidative stress state in ovaries. As a result, a decreased *GPD1L* level was observed (Figure S3C). Next, by using Pearson correlation coefficient testing, we observed a positive correlation between *GPD1L* levels and AMH (Figure 3C), while a negative correlation with female age (Figure 3D).

Additionally, *GPD1L* expression levels positively correlated with the key metabolites in the glycerophospholipid metabolism pathway (Figure 3E). To further validate our speculation that severe impairment of the glycerophospholipid metabolism may result from *GPD1L* downexpression, we downregulated *GPD1L* expression in KGN cells with small interfering RNA (siRNA) transfection. After validating the siRNA knockdown (KD) efficiencies by RT‐qPCR and WB (Figure 3F–H), we performed the quantitative lipidomics analysis of siRNA‐transfected KGN cells. The PCA and OPLS‐DA first showed a clustering between negative control (NC) and siR‐GPD1L groups (Figure S4A,B). In total, 25 up‐regulated metabolites and 134 down‐regulated metabolites were identified between the two groups (Figure S4C). Then, categorisation analysis demonstrated that 74.84% of the differential metabolites are glycerophospholipids (Figure 3I), and further, the bubble diagram and heatmap revealed the down‐expression of metabolites in the glycerophospholipids pathway following *GPD1L*‐KD (Figure 3J,K). In accordance with this, the KEGG pathway analysis indicated that the glycerophospholipids metabolism is the most enriched pathway (Figure 3L). Besides, as another important component of phospholipids and downstream metabolites of phospholipid metabolism, respectively, the sphingolipid metabolism pathway and arachidonic acid metabolism pathway were also enriched in our study (Figure 3L). Taken together, our data validated the downregulation of *GPD1L* and its mediated glycerophospholipids metabolism pathway dysfunction in granulosa cells of DOR patients, which is highly associated with the decline of ovarian function.

### 2.4 Downregulated *GPD1L* Induces Cell Apoptosis Under Oxidative Stress and Inhibits Cell Proliferation

**FIGURE 4:**
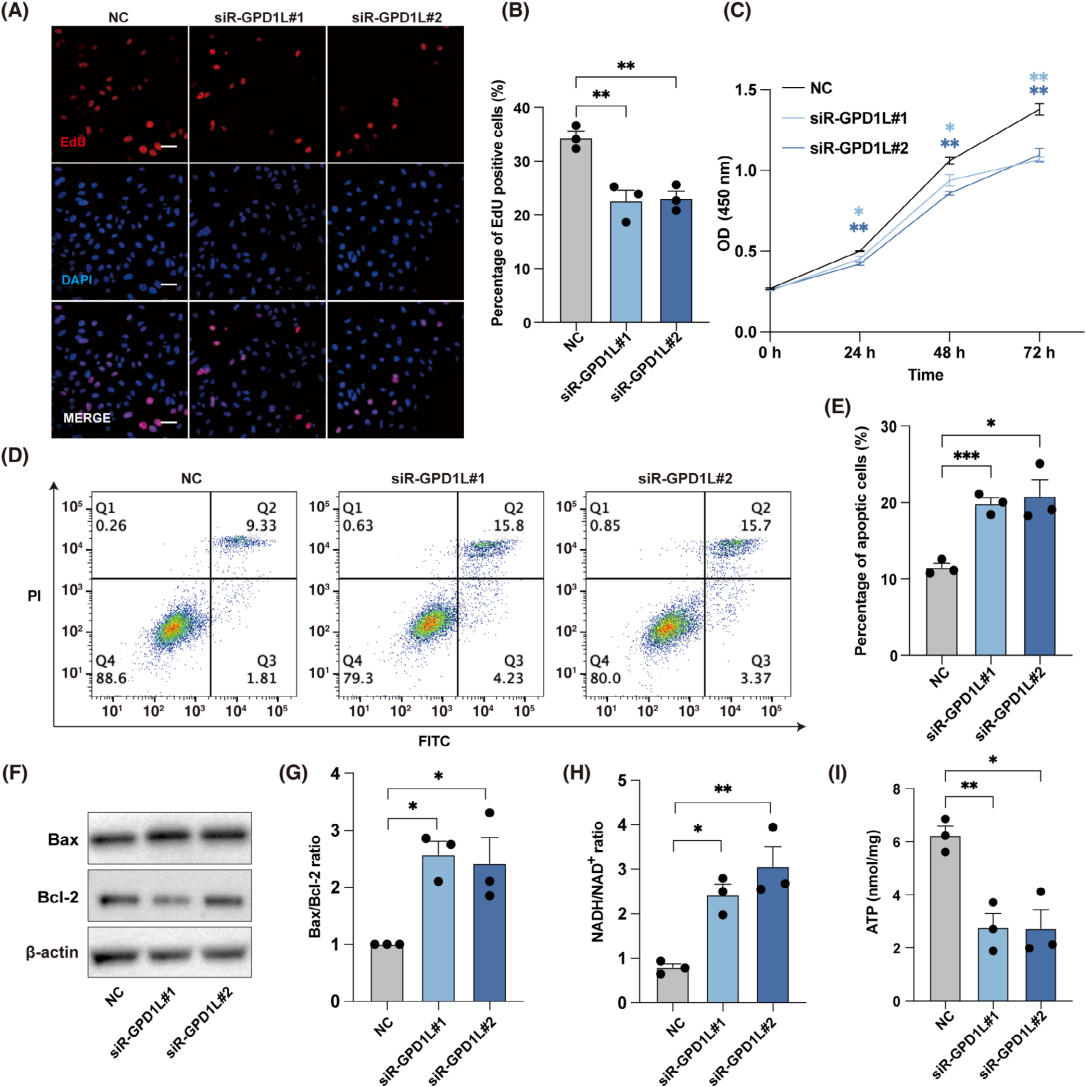
*GPD1L* Knockdown inhibits cell proliferation and induces cell apoptosis. (A) Representative images of proliferative cells in *GPD1L*‐KD KGN cells reflected by EdU staining. Scale bars, 50 μm. (B) Statistics of the quantification of EdU‐positive KGN cells. (*n* = 3 independent experiments per group). (C) CCK 8 assay of *GPD1L*‐KD KGN cells after culturing for 24, 48, and 72 h. (*n* = 3 independent experiments per group). (D) The flow cytometry analysis of cell apoptosis of *GPD1L*‐KD KGN cells after being treated with 300 μM H_2_O_2_ using Annexin V‐FITC/PI staining. (E) Quantification of the apoptotic cells identified by Annexin V‐FITC/PI staining and flow cytometry analysis. (*n* = 3 independent experiments per group). (F) Representative western blotting images showing Bax and Bcl‐2 protein levels in *GPD1L*‐KD KGN cells. (G) Quantification of the ratio of Bax/Bcl‐2 protein levels. (*n* = 3 independent experiments per group, protein levels were normalised to β‐actin). (H) Quantification of NADH/NAD^+^ ratio in *GPD1L*‐KD KGN cells. (*n* = 3 independent experiments per group). (I) ATP detection of *GPD1L*‐KD KGN cells. (*n* = 3 independent experiments per group). All data were presented as mean ± SEM. *p < 0.05, **p < 0.01, ***p < 0.001.

To gain insights into the potential roles of *GPD1L*, we downregulated *GPD1L* gene expression in KGN cells with siRNA transfection. The cell proliferation ability was first determined, in which the EdU assay demonstrated a significantly lower percentage of proliferative cells in *GPD1L*‐KD groups, and a lower number of cells was determined by the CCK8 assay after culturing for 24, 48, and 72 h (Figure 4A–C). Next, Annexin V‐FITC/PI labeling and flow cytometry analysis were performed to investigate the role of *GPD1L* in reducing cell apoptosis under oxidative stress. We supplemented H_2_O_2_ into the cell culture medium after the siRNA transfection to simulate the ovarian oxidative stress state. *GPD1L*‐KD groups showed a significantly higher proportion of apoptotic cells compared to the NC group (Figure 4D,E). Moreover, the WB analysis showed elevated protein levels of B‐cell lymphoma 2 associated X‐protein (Bax) and reduced levels of B‐cell lymphoma 2 (Bcl‐2) in *GPD1L*‐KD groups, which are classical apoptosis‐related proteins (Figure 4F,G). Since the process by which GPDH oxidises NADH by converting dihydroxyacetone phosphate (DHAP) to glycerol‐3‐phosphate (G3P) is an NAD^+^‐dependent reversible reaction [33, 34], correspondingly a significantly increased NADH/NAD^+^ ratio was observed in the *GPD1L*‐KD groups (Figure 4H). In addition, we also detected the concentration of cellular ATP, in which a decreased ATP production level was identified in *GPD1L‐*KD KGN cells (Figure 4I). Taken together, we validated that downregulation of *GPD1L* could inhibit cell proliferation, increase cell apoptosis under oxidative stress, and lead to cell energy metabolism disorder.

### 2.5 Downregulated *GPD1L* Leads to Abnormal Mitochondrial Morphology and Impairs Mitochondrial Function

Given that glycerophospholipids are the most abundant phospholipids in the membranes of mammalian cells and are pivotal for the function and structure of mitochondria [28, 35], we further looked into the link between *GPD1L* downregulation and mitochondrial functions.

**FIGURE 5:**
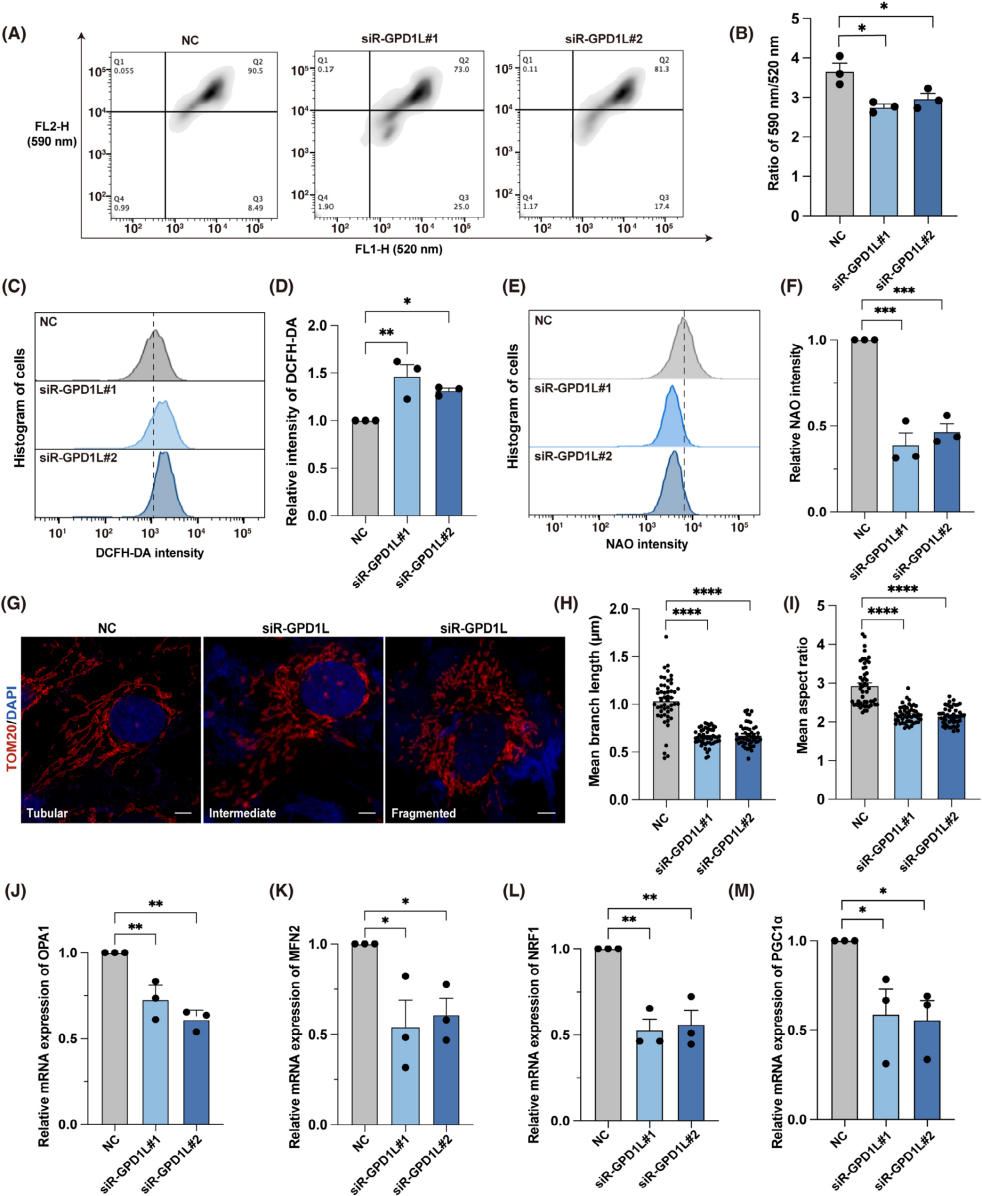
*GPD1L* knockdown results in mitochondrial dysfunction of granulosa cells. (A) Flow cytometry analysis of mitochondrial membrane potential of *GPD1L*‐KD KGN cells using JC‐10 probe. (B) Quantification of the 590/520 nm intensity ratio identified in JC‐10 flow cytometry analysis in Figure 5A. (*n* = 3 independent experiments per group). (C) DCFH‐DA staining and flow cytometry analysis of the cellular ROS levels in *GPD1L*‐KD KGN cells. (D) Quantification of the DCFH‐DA intensity identified in flow cytometry analysis in Figure 5C. (*n* = 3 independent experiments per group). (E) NAO staining and flow cytometry analysis of the mitochondrial mass of *GPD1L*‐KD KGN cells. (F) Quantification of the NAO intensity identified in flow cytometry analysis in Figure 5E. (*n* = 3 independent experiments per group). (G) Representative images of mitochondrial morphology of *GPD1L*‐KD KGN cells. Mitochondria were labelled by TOM20. Scale bars, 5 μm. (H) Quantification of the mitochondrial mean branch length (μm). (*n* = 40 cells in 3 independent experiments per group). (I) Quantification of the mitochondrial mean aspect ratio. (*n* = 50 cells in 3 independent experiments per group). (J) The expression levels of *OPA1* in *GPD1L*‐KD KGN cells. (*n* = 3 experiments per group, gene expression was normalised to *ACTB*). (K) The expression levels of *MFN2* in *GPD1L*‐KD KGN cells. (*n* = 3 experiments per group, gene expression was normalised to *ACTB*). (L) The expression levels of *NRF1* in *GPD1L*‐KD KGN cells. (*n* = 3 experiments per group, gene expression was normalised to *ACTB*). (M) The expression levels of *PGC1α* in *GPD1L*‐KD KGN cells. (*n* = 3 experiments per group, gene expression was normalised to *ACTB*). All data were presented as mean ± SEM. *p < 0.05, **p < 0.01, ***p < 0.001, ****p < 0.0001.

In this, we initially assessed the mitochondrial membrane potential (MMP) and reactive oxygen species (ROS) levels using the flow cytometry analysis of JC‐10 and DCFH‐DA staining respectively. The *GPD1L*‐KD groups showed lower MMP and increased ROS levels (Figure 5A–D), suggesting the deterioration of mitochondrial function. Secondly, as shown in Figure 5E,F, declined mitochondrial mass was identified in the *GPD1L*‐KD groups, as determined by flow cytometry analysis using Acridine Orange 10‐Nonyl Bromide (NAO, a cardiolipin‐binding fluorescence dye), indicating a decreased level of cardiolipin in the mitochondrial inner membrane. To further investigate the alterations in mitochondrial morphology, we subsequently labelled the mitochondrial outer membrane with TOM20. It was found that *GPD1L* downregulation resulted in fragmented mitochondria (Figure 5G) with an obvious decrease in mitochondrial mean branch length and mean aspect ratio (Figure 5H,I).

Since the observations above suggested an impaired mitochondrial biogenesis process in *GPD1L*‐KD cells, we further explored the underlying mechanisms of mitochondrial dysfunction caused by *GPD1L* downregulation. Mitochondria are dynamic organelles that continuously undergo fission and fusion to sustain normal mitochondrial function [36, 37]. The downregulation of the genes critical to mitochondrial fusion (*OPA1*, *MFN2*) was identified in our study (Figure 5J,K), suggesting the imbalance in mitochondrial fission–fusion in *GPD1L*‐KD groups. Furthermore, our results showed that the expression level of nuclear respiratory factor‐1 (*NRF1*) and peroxisome proliferator‐activated receptor gamma coactivator 1‐alpha (*PGC1α*), genes that are highly related to the regulation of biogenesis and function of mitochondria [38, 39], were significantly decreased in KGN cells transfected with *GPD1L* siRNA (Figure 5L,M). Taken together, we demonstrated that downregulated *GPD1L* could lead to abnormal mitochondrial morphology and impaired mitochondrial function by affecting mitochondrial biogenesis, thereby resulting in the dysfunction of ovarian granulosa cells.

### 2.6 *Gpd1l* Downregulation in Mouse Ovary Induces Follicular Atresia and Impairs Oocyte Quality

**FIGURE 6:**
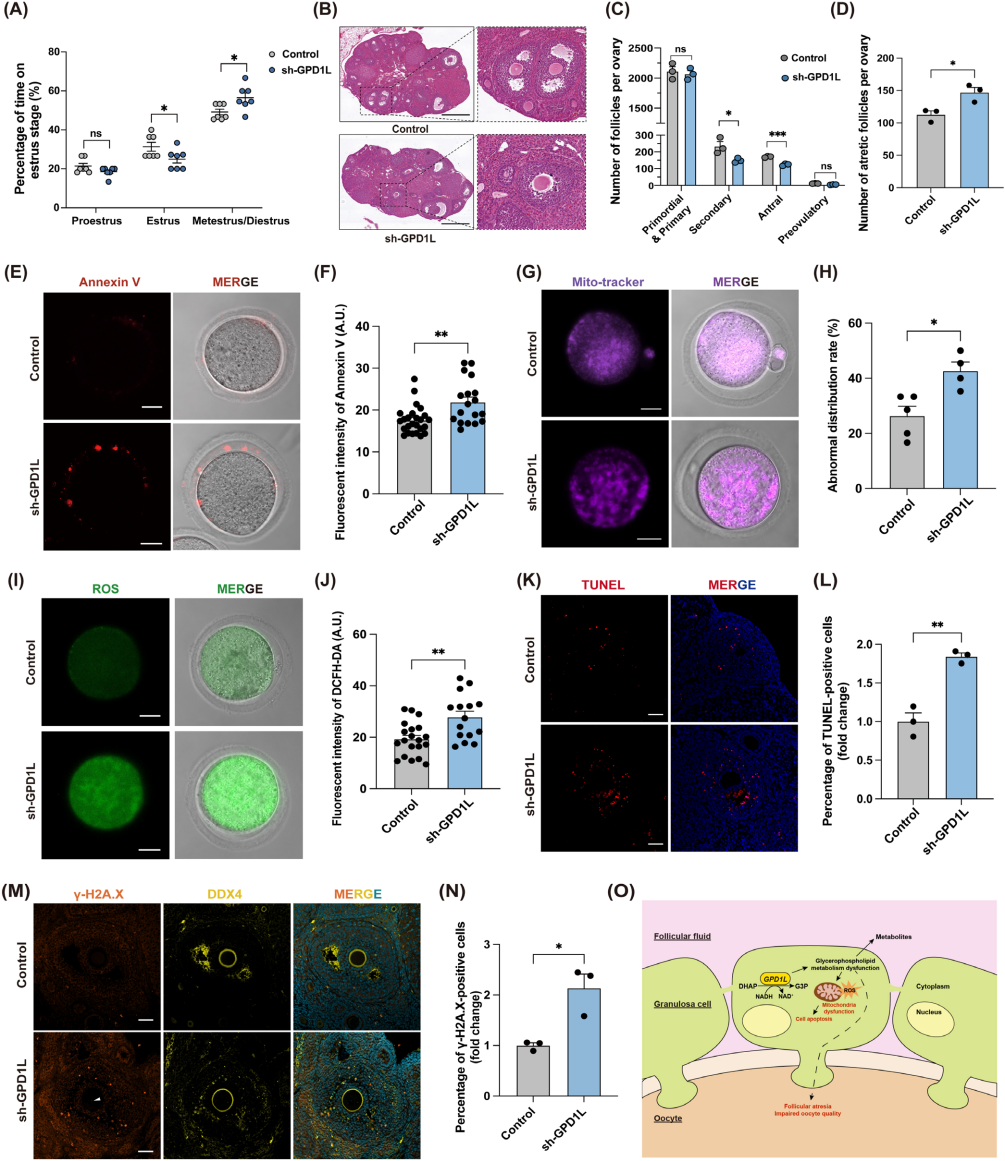
The impacts of *Gpd1l* knockdown on oocyte quality in mouse ovaries. (A) Percentage of time that sh‐GPD1L and control group mice spent in major estrus cycle stages after ovarian in situ microinjection. (*n* = 7 mice per group). (B) Representative H&E staining images of mouse ovaries in the sh‐GPD1L and control groups. Scale bars, 500 μm. (C) Quantification of follicles in different developmental stages. (*n* = 3 mice per group). (D) The number of atretic follicles in the sh‐GPD1L and control groups. (*n* = 3 mice per group). (E) Representative images of Annexin V staining of the sh‐GPD1L group and control group oocytes. Scale bars, 20 μm. (F) Quantification of Annexin V fluorescence intensity in the sh‐GPD1L group and control group oocytes. (*n* = 25 oocytes for the control group and *n* = 18 oocytes for the sh‐GPD1L group). (G) Representative images of mitochondria distribution of sh‐GPD1L group and control group oocytes. Scale bars, 20 μm. (H) Quantification of abnormal mitochondria distribution rate of two groups oocytes. (*n* = 5 mice for the control group and *n* = 4 mice for the sh‐GPD1L group). (I) Representative images of DCFH‐DA staining of sh‐GPD1L group and control group oocytes. Scale bars, 20 μm. (J) Quantification of DCFH‐DA fluorescence intensity in the sh‐GPD1L group and control group oocytes. (*n* = 20 oocytes for the control group and *n* = 15 oocytes for the sh‐GPD1L group). (K) Representative images of apoptotic cells in mice ovaries in the sh‐GPD1L group and control group reflected by TUNEL assay. Scale bars, 50 μm. (L) Quantification of the relative percentage of TUNEL‐positive granulosa cells. (*n* = 3 mice per group). (M) Representative images of γ‐H2A.X immunofluorescence staining of mice ovaries in the sh‐GPD1L group and control group. The white arrowhead indicated the DNA damage foci in oocytes. Oocytes were labelled by DDX4. Scale bars, 50 μm. (N) Quantification of the relative percentage of γ‐H2A.X‐positive granulosa cells. (*n* = 3 mice per group). (O) The mechanisms diagram of GPD1L‐mediated glycerophospholipid metabolism dysfunction in DOR patients. All data were presented as mean ± SEM. *p < 0.05, **p < 0.01, ***p < 0.001.

Following an investigation into the potential mechanisms of *GPD1L* in ovarian granulosa cells, we subsequently examined its effects on female fertility. In this, 3‐month‐old mice received in situ ovarian microinjection with Mouse_Gpd1l‐shRNA‐GPAAV‐Egfp to stably downregulate the expression of *Gpd1l* (sh‐GPD1L group). Firstly, to investigate whether *Gpd1l* down‐expression in mouse ovaries would induce follicular atresia and cause impaired follicular development, we first detected the estrous cycle of mice in two groups by microscopic analysis of vaginal smears after validating the efficiencies of *Gpd1l*‐KD in mice ovaries (Figure S5A,B). The control group mice displayed regular estrous cycles, while irregular estrous cycles were found in the sh‐GPD1L group mice (Figure S5C), exhibiting fewer estrus stages and more metestrus/diestrus stages (Figure 6A). Next, the morphological changes of mouse ovaries were detected by haematoxylin and eosin (H&E) staining. The number of follicles of different developmental stages was counted in each group respectively. The results showed a decline in the number of growing follicles in the ovaries of the sh‐GPD1L group, while no significance was found in primordial and primary follicles, indicating a disabled follicular development in *Gpd1l*‐downregulated ovaries (Figure 6B,C). Besides, we also counted the atretic follicles in the ovaries and found an increased atretic follicle number in the sh‐GPD1L group (Figure 6D). Oocyte quality can serve as an indicator for evaluating female ovarian function [40]. In this, to detect the alterations in oocyte quality caused by *Gpd1l* down‐expression, we performed superovulation on mice 1 week after the AAV microinjection. Annexin‐V staining was used as an indicator to assess the apoptosis level of oocytes. As demonstrated in Figure 6E,F, oocytes in the sh‐GPD1L group displayed increased fluorescence signal intensity, suggesting the initiation of early apoptosis of oocytes. We also examined the mitochondrial function of oocytes in light of their vital role as specialised organelles that are key to oocyte energy production and developmental competence [41]. Labelled by Mito‐tracker, mitochondria were more aggregated in the oocyte cytoplasm of the sh‐GPD1L group (Figure 6G,H). As mitochondrial dysfunction can lead to increased intracellular ROS production [42], we detected the ROS level of the oocytes as well, in which excessive ROS was observed in the sh‐GPD1L group oocytes (Figure 6I,J). Moreover, a lower MMP was exhibited in the sh‐GPD1L group oocytes, stained by JC‐10 probe (Figure S5D,E). Except for the tests on indicators of oocyte quality, we also detected the function of granulosa cells in mice ovaries. We observed an increased proportion of apoptotic granulosa cells and fewer proliferative cells among the large growing follicles in the sh‐GPD1L group by TUNEL assay (Figure 6K,L) and immunofluorescent staining of Ki67 (Figure S5F,G) respectively. In addition, DNA damage can be induced by excessive ROS and is one of the major triggers of cell apoptosis [43]. In our study, the occurrence of DNA damage both in granulosa cells and oocytes was observed in the sh‐GPD1L group labelled by γ‐H2A.X staining (Figure 6M,N). Altogether, all the data above indicated the aggravated damage in granulosa cells as well as increased follicular atresia and impaired oocyte quality induced by *GPD1L* downregulation via mitochondrial dysfunction and excessive accumulation of ROS.

## 3 Discussion

DOR is a pathological process involving multiple etiologies, which is essentially the decline of oocyte quantity and quality that will inevitably lead to female infertility and seriously affect female reproductive health and quality of life [44, 45]. An in‐depth understanding of the metabolic status of FF will help us reveal the important biological physiology processes and identify potential biomarkers and targets of ovarian aging. Despite several metabolomics analyses that have revealed the association between metabolic profile alterations of FF and the occurrence of DOR, their specific underlying mechanisms still remain unclear [16, 17, 46]. Pseudotargeted metabolomics based on UHPLC‐TQMS merges the advantages of untargeted and targeted metabolomics with higher sensitivity, higher specificity, and excellent quantification ability [47]. In this current study, by utilising the pseudotargeted metabolomics technology, we identified a downregulated glycerophospholipid metabolism pathway in the FF of women with DOR, correlated with the reduced *GPD1L* expression levels in granulosa cells. Moreover, we further validated that the downregulation of *GPD1L* participates in ovarian aging by inducing granulosa cell apoptosis and mitochondrial dysfunction, as well as inducing follicular atresia and impairing oocyte quality, which provides new insights into the mechanism of ovarian aging (Figure 6O).

Glycerophospholipids are the most abundant lipids of mammalian cell membranes and can further be divided into phosphatidic acid (PA), phosphatidylethanolamine (PE), phosphatidylcholine (PC), phosphatidylinositol (PI), and phosphatidylserine (PS) based on the structures of polar head groups [48]. Beyond their role as crucial components of cell membranes, they are essential for various biological processes, such as mitochondrial activity, cellular signalling pathway transduction, cell apoptosis, and membrane protein localization [18, 49]. Glycerophospholipid metabolism is one of the important parts of cellular metabolism and requires the participation of multiple key enzymes. *GPD1L* encodes GPDH, the key enzyme in the glycerophospholipid metabolism pathway. With the help of the transcriptomic data from the database, we found that *GPD1L* was dynamically expressed in oocytes and granulosa cells of all five follicular developmental stages with higher expression levels than *GPD1*, indicating its potential role in follicular development and oocyte maturation. Our current study further identified decreased glycerophospholipid‐associated metabolites PA, PC, LysoPC, and LysoPE levels in FF of DOR patients, as well as downregulated *GPD1L* levels in granulosa cells, which were positively correlated with female ovarian function. In addition, we further confirmed the correlation between *GPD1L* and the downregulation of the glycerophospholipid pathway through quantitative lipidomics analysis of *GPD1L*‐KD KGN cells. These results suggested the presence of *GPD1L‐*mediated glycerophospholipid metabolism dysfunction in DOR patients and may serve as a potential diagnostic biomarker.

Follicular development and oocyte maturation are closely associated with granulosa cell function and highly rely on metabolic and energy supports from them [11]. Follicular atresia is considered the main reason for accelerated follicular consumption during ovarian aging and mainly results from granulosa cell apoptosis [50]. Hence, we looked into the association between *GPD1L* downregulation and alterations in granulosa cell function. We first identified an increased proportion of apoptotic KGN cells after *GPD1L* knockdown utilising in vitro validation experiments. Then, we further focused on the alterations in granulosa cell functions in vivo. As a result, increased apoptotic granulosa cells and decreased proliferative cells were observed in the ovaries of the sh‐GPD1L group mice. We also found the occurrence of DNA damage in ovarian granulosa cells as well as in oocytes. Besides, energy metabolism is one of the key elements affecting cell function, and the NADH/NAD^+^ redox balance is fundamental to cellular energy production [51]. The GPDH‐catalysed conversion of DHAP to G3P involves the oxidation of NADH to NAD^+^, after which the electrons from cytosolic NADH are transferred to the electron transport chain (ETC) via the G3P shuttle process for ATP synthesis [24]. Correspondingly, *GPD1L*‐KD KGN cells displayed an increased NADH/NAD^+^ ratio and decreased ATP levels, representing an impaired cellular energy metabolism of granulosa cells. Collectively, our results suggested aggravated damage in granulosa cells and a degenerated microenvironment for follicular development, which is consistent with the increased follicular atresia we observed in mouse ovaries.

It has been well established that mitochondria are critically important to ovarian function and reproductive longevity [52]. Since mitochondria are important organelles that maintain normal biological functions of the cells, we further investigated the link between the mitochondrial function of granulosa cells and *GPD1L* downregulation. Mitochondria serve as the primary source of ROS production and are also susceptible to ROS‐induced cellular damage [53]. Bcl‐2 is an anti‐apoptotic protein that could bind to and interact with Bax, thereby preventing the outer mitochondrial membrane (OMM) permeabilisation and inhibiting cell apoptosis [54]. A previous study has indicated that excessive ROS may induce mitochondrial damage by regulating Bax/Bcl‐2 expression [55]. Here, we demonstrated the abnormal ROS accumulation and increased apoptotic index of Bax/Bcl‐2 in *GPD1L*‐downregulated KGN cells, suggesting the degeneration of mitochondrial function. PE, PC, CL, and PI constitute the main lipid components of the mitochondrial membrane, and the continuous supply and exchange of lipids are required for maintaining mitochondrial membrane integrity and overall cellular function [56]. CL is primarily located in the inner mitochondrial membrane (IMM) and is critical to membrane structural integrity and stabilisation of the electron transfer complex [48, 57]. A decreased mitochondria mass was observed in *GPD1L*‐KD KGN cells by utilising NAO staining, a probe specifically binding with CL in the IMM. Besides, quantitative lipidomics analysis of *GPD1L*‐KD KGN cells demonstrated the decreased intensities of several glycerolphospholipid‐associated metabolites PC, PE, PC‐O, and PI. These clues indicated an imbalanced cellular lipid component caused by *GPD1L* downregulation. Mitochondrial morphology is crucial for mitochondrial function, and growing evidence has shown that the elimination of the phospholipid components in the mitochondria membrane could result in abnormal mitochondrial morphology [58, 59]. It has also been demonstrated that excessive cellular oxidative stress could cause mitochondrial fragmentation [60]. Accordingly, we identified fragmented mitochondria of KGN cells resulting from *GPD1L* downregulation. Mitochondria constantly undergo dynamic fission and fusion to maintain normal structure and functions, in which fusion is modulated mainly by mitofusin proteins MFN1/2 and GTPase OPA1 [36]. It was demonstrated that OPA1 and cardiolipin interactions drive mitochondria membrane fusion in cells [61] and mitochondria were more fragmented in megakaryocytes derived from *Mfn2*‐knockout mice [62]. In accordance with this, we found decreased expression levels of *OPA1* and *MFN2* after *GPD1L* downregulation, indicating the defect of mitochondrial fusion in granulosa cells. As key regulators of mitochondrial biogenesis, the important role of the PGC1‐α/NRF1 signalling pathway in mitochondrial function has been confirmed in several studies [63, 64]. Currently, we detected that the knockdown of *GPD1L* has an impact on the expression of *PGC1‐α* and *NRF1*, thereby disrupting mitochondrial dynamic homeostasis.

Moreover, it is well known that the granulosa cell function and the metabolic components of follicular fluid are closely related to oocyte quality [11]. The levels of PE, LPE, and PC‐O in FF were found to be positively correlated with clinical indicators including oocytes retrieved, MII oocytes, and high‐quality embryos [65]. It was also reported that LPC is closely related to apoptosis and is pivotal to the growth of follicles and the maturation of oocytes [66, 67]. Considering that we found granulosa cell dysfunction through validation experiments and decreased LPE, LPC, PE, and PC levels by metabolomics, we further investigated the follicular development and oocyte quality in mouse ovaries down expressing *Gpd1l*. In this regard, irregular estrous cycles and a decreased number of growing follicles in mice ovaries suggested disorders in follicular development. Mitochondria keep the balance between ATP production and ROS generation, having a critical role in oocyte maturation, fertilisation, and early embryo development [68]. Here, we identified deteriorated oocyte mitochondrial function indicated by abnormal mitochondria distribution and decreased MMP, as well as aberrant ROS accumulation in oocytes from the sh‐GPD1L group. Besides, Annexin‐V staining revealed the occurrence of early apoptosis of sh‐GPD1L group oocytes. Overall, all these clues demonstrated the impacts that the *GPD1L*‐mediated glycerophospholipid metabolism dysfunction has on female fertility.

## 4 Conclusion

In conclusion, our study demonstrated the dysfunction of *GPD1L*‐mediated glycerophospholipid metabolism in patients with DOR. We revealed that downregulated *GPD1L* expression in granulosa cells induces cell apoptosis, leads to mitochondrial dysfunction via the disruption of mitochondrial biogenesis, as well as increases follicular atresia and impairs oocyte quality. Whereas the validity of those differential metabolites we identified in this study as clinical diagnostic biomarkers still needs to be confirmed in large‐scale studies. Hence, we proposed that *GPD1L* and the key metabolites within the glycerophospholipid metabolism pathway may act as promising new biomarkers for DOR diagnosis and offered a fresh perspective and a new theoretical basis for the pathogenesis of DOR.

## Author Contributions

Xiaokui Yang and Long Yan designed and supervised the study. Jiaqi Wu and Xuehan Zhao conducted the validation experiments and prepared the figures and tables. Xuehan Zhao performed the pseudotargeted metabolic data analysis. Cong Wang and Yichang Tian helped collect the clinical samples. Wan Tu and Qiqian Wu helped with the animal experiments. Jiaqi Wu wrote the original manuscript with inputs from all co‐authors. Ying Fang, Long Yan, and Xiaokui Yang helped edit and revise the manuscript. All the authors read the manuscript and approved the final version.

## Ethics Statement

This study was approved by The Ethics Committee of the Beijing Obstetrics and Gynecology Hospital, Capital Medical University (Code: 2023‐KY‐097‐01). Informed written consent was obtained from all participants.

## Conflicts of Interest

The authors declare no conflicts of interest.

## Supporting information


Figure S1.
Figure S2.
Figure S3.
Figure S4.
Figure S5.

Table S1.

Table S2.

Table S3.

**Data S1.** Materials and Methods.

## Data Availability

Data sharing is not applicable to this article as no new data were created or analyzed in this study.
