# Supplementary material for: GPD1L‐Mediated Glycerophospholipid Metabolism Dysfunction in Women With Diminished Ovarian Reserve: Insights From Pseudotargeted Metabolomic Analysis of Follicular Fluid
Source: Cell Prolif. 2025 Mar 20;58(9):e70024. doi: 10.1111/cpr.70024 (PMC12414641; doi:10.1111/cpr.70024)
Supplement: Supplementary file 1 — Figure S1. Figure S2. Figure S3. Figure S4. Figure S5. [file CPR-58-e70024-s003.docx]

**Supplementary Figures**

**FIGURE S1** The quality control of metabolome data of follicular fluids based on QC samples. (A) The percentage of the data type of the follicular fluids samples from different groups. (B) Diagram showing the ratio of each equal range of MS intensity after equal division among different groups. (C) Correlation of the mean values of MS intensity of each feature in experimental samples and in QC samples evaluated by Pearson’s coefficient. A, young NOR group; B, aged NOR group; C, young DOR group; D, aged DOR group.

**
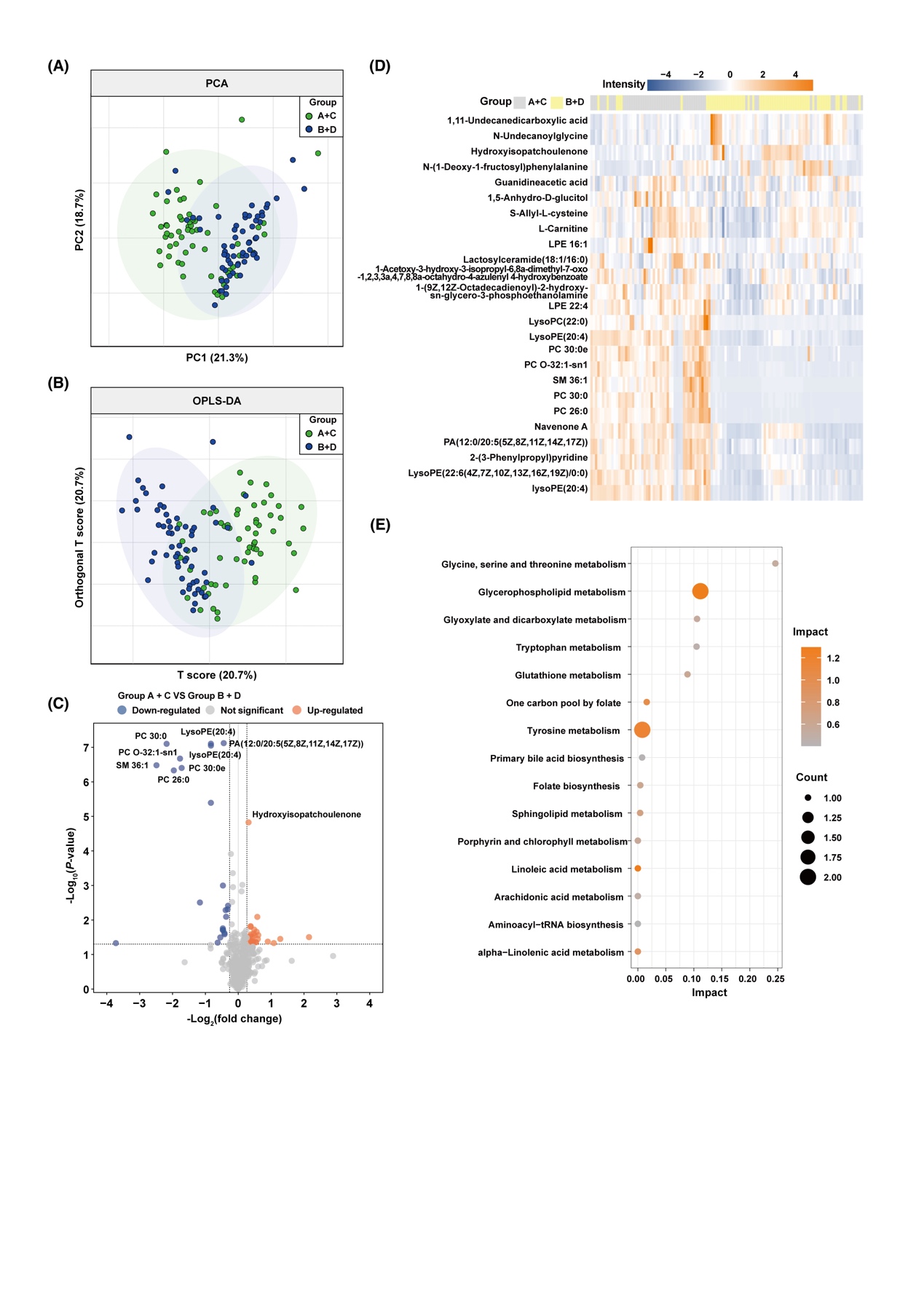
**

**FIGURE S2** The metabolic profiles of follicular fluids between young and aged groups. (A) The scores scatter plot of PCA based on the data of follicular fluid samples between young and aged groups. (B) The scores scatter plot of OPLS-DA based on the data of follicular fluid samples between young and aged groups. (C) Volcano plot showing differential metabolites between young and aged groups. Orange dots for up-regulated and blue dots for down-regulated in the aged group. (D) Heatmap showing the levels of the representative differential metabolisms in young and aged groups. The color bar from blue to orange indicates low to high levels of metabolisms. (E) Bubble plot showing the representative KEGG pathways enriched with the differential metabolisms. The size of the bubble represents the amounts of metabolites enriched in the pathways.

**FIGURE S3** Expression levels of GPD1, GPD1L and GPD2 during folliculogenesis. (A) Box plots of the dynamic expression of GPD1, GPD1L and GPD2 in oocyte and granulosa cells during folliculogenesis respectively. (B) Representative images of GPD1L expression in the ovaries of 3-month-old and 10-month-old mice. Scale bars, 50 μm. (C) The expression levels of *GPD1L* in KGN cells treated with H_2_O_2_. (n = 3 experiments per group, gene expression was normalized to *ACTB*). All data were presented as mean ± SEM. ***P* < 0.01.

**
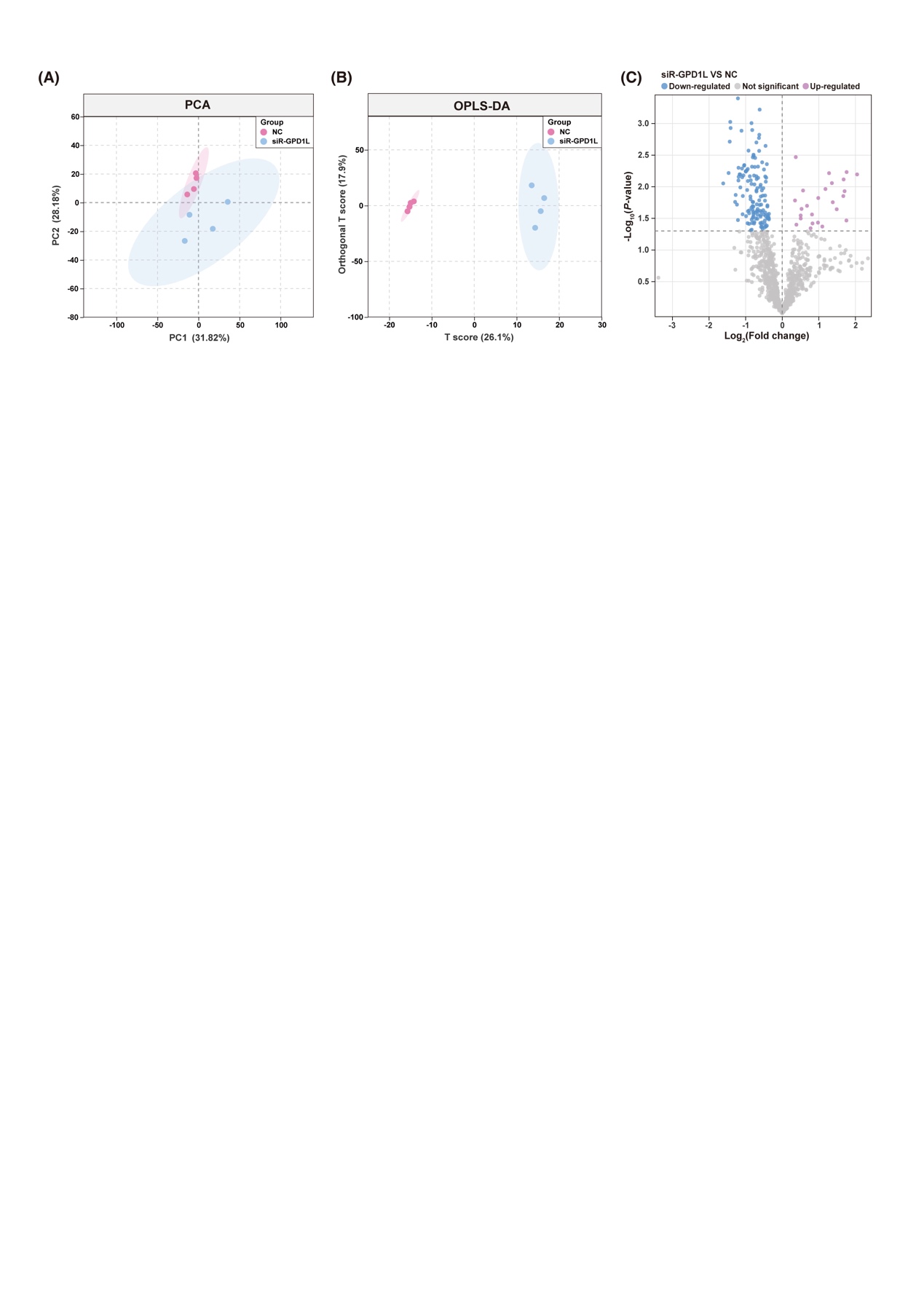
**

**FIGURE S4** The different metabolic profiles of cell samples between NC and siR-GPD1L groups. (A) The scores scatter plot of PCA based on the data of cell samples between NC and siR-GPD1L groups. (B) The scores scatter plot of OPLS-DA based on the data of cell samples between NC and siR-GPD1L groups. (C) Volcano plot showing differential metabolites between NC and siR-GPD1L groups. Purple dots for up-regulated and blue dots for down-regulated in the siR-GPD1L group.

**
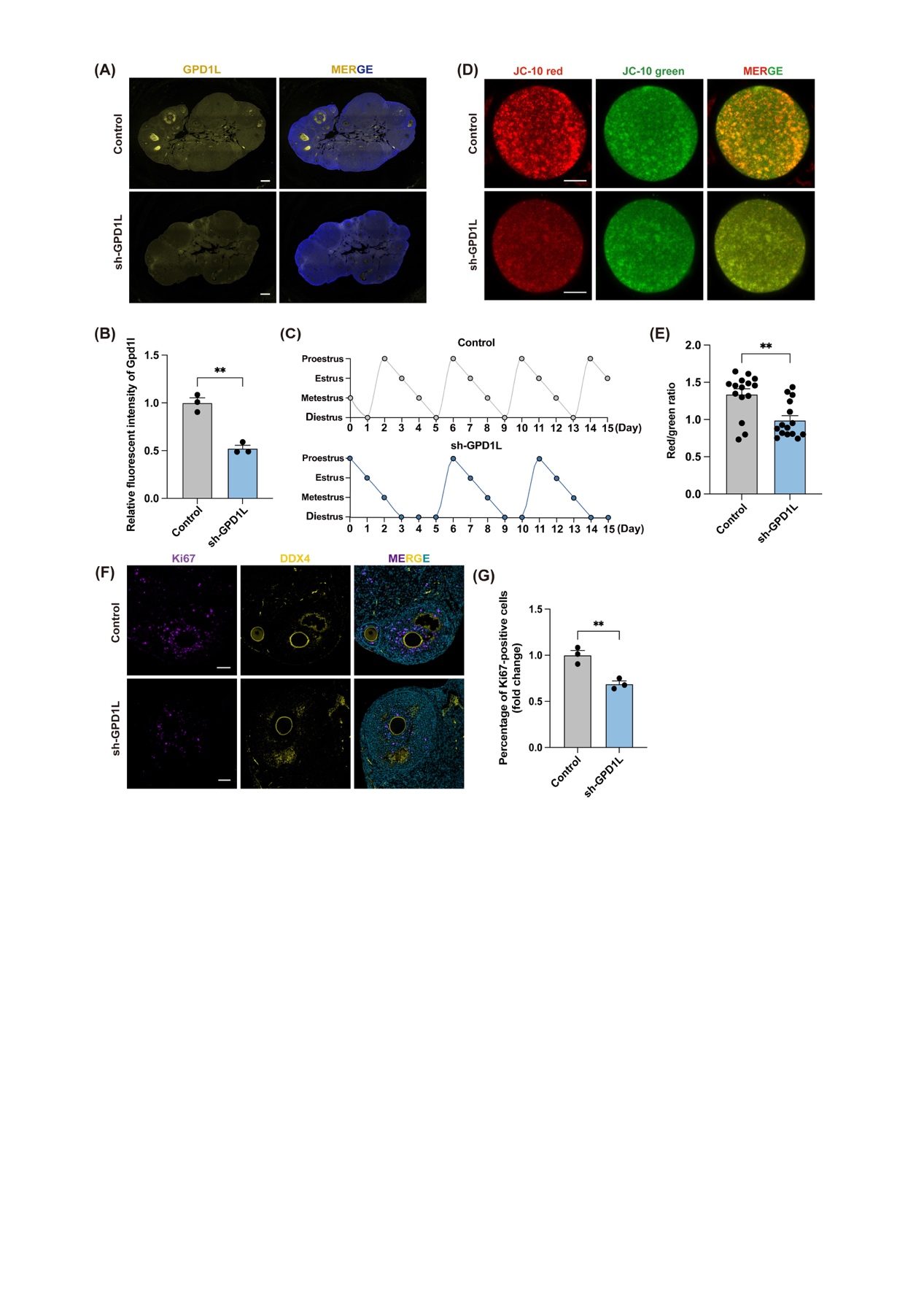
**

**FIGURE S5** The impacts of *Gpd1l* knockdown on oocyte quality in mouse ovaries. (A) Representative images of Gpd1l immunofluorescence staining of mice ovaries in the sh-GPD1L and control groups. Scale bars, 200 μm. (B) Quantification of relative Gpd1l fluorescence intensity of mice ovaries in the sh-GPD1L and control groups (n = 3 mice per group). (C) Representative graphs showing the estrous cycle of mice in the sh-GPD1L and control groups. (D) Representative images of the MMP of sh-GPD1L group and control groups oocytes. Scale bars, 20 μm. (E) Quantification of the JC-10 red/green fluorescence intensity ratio in the sh-GPD1L group and control group oocytes. (n = 15 oocytes per group). (F) Representative images of Ki67 immunofluorescence staining of mice ovaries in the sh-GPD1L and control groups. Scale bars, 50 μm. (G) Quantification of the relative percentage of Ki67-positive granulosa cells. (n = 3 mice per group). All data were presented as mean ± SEM. **P < 0.01.
