# Supplementary material for: GPD1L‐Mediated Glycerophospholipid Metabolism Dysfunction in Women With Diminished Ovarian Reserve: Insights From Pseudotargeted Metabolomic Analysis of Follicular Fluid
Source: Cell Prolif. 2025 Mar 20;58(9):e70024. doi: 10.1111/cpr.70024 (PMC12414641; doi:10.1111/cpr.70024)
Supplement: Supplementary file 2 — Table S1. [file CPR-58-e70024-s001.docx]

**Table S1. Demographics and characteristics of the study population**

| **Covariates** | **Young NOR**  **(Group A, n=30)** | **Aged NOR**  **(Group B, n=30)** | **Young DOR**  **(Group C, n=29)** | **Aged DOR**  **(Group D, n=30)** | ***P-value*** |
| --- | --- | --- | --- | --- | --- |
| Age (y) | 32 (30, 33) | 38 (37, 41) ^a^ | 31 (29, 33) ^b^ | 37 (35,39) ^a,c^ | <0.001 |
| Infertile period (y) | 3 (2, 4) | 2 (1.5, 5) | 3 (2, 5.5) | 3 (2, 6) | 0.351 |
| Infertility type |  |  |  |  | 0.008 |
| Primary infertility | 8 | 20 ^a^ | 9 ^b^ | 12 ^b^ |  |
| Secondary infertility | 22 | 10 | 20 | 18 |  |
| Body mass index (kg/m^2^) | 21.60  (20.50, 24.20) | 23.30  (20.60, 26.40) | 21.00  (19.85, 24.80) | 22.60  (20.70, 24.49) | 0.457 |
| Basal FSH (mIU/L) | 6.34  (5.46, 7.56) | 6.29  (5.66, 7.66) | 7.98  (6.63, 10.08) ^a,b^ | 8.42  (7.17, 10.30) ^a,b^ | <0.001 |
| Basal LH (mIU/mL) | 4.34  (2.79, 6.39) | 3.43  (2.53, 4.42) | 4.04  (2.97, 5.35) | 3.75  (2.59, 4.53) | 0.244 |
| Basal FSH/LH | 1.66  (1.08, 2.59) ^b^ | 2.01  (1.41, 2.72) | 2.21  (1.60, 2.64) | 2.54  (2.04, 3.51) | 0.011 |
| Basal E_2_ (pg/mL) | 40.92  (33.20, 51.26) | 35.35  (30.77, 52.40) | 50.54  (37.90, 64.56) | 42.76  (26.07, 60.01) | 0.234 |
| AMH (ng/mL) | 3.16  (2.05, 6.92) | 2.53  (1.87, 4.36) | 1.10  (0.62, 2.60) ^a,b^ | 0.67  (0.42, 1.66) ^a,b^ | <0.001 |
| TSH (mIU/L) | 2.27  (1.67, 2.77) | 1.72  (1.32, 2.35) | 1.77  (1.57, 2.73) | 2.86  (1.61, 3.80) | 0.054 |
| Antral follicle count (n) | 12 (10, 15) | 9 (8, 14) | 4 (4, 8) ^a,b^ | 3 (2, 6) ^a,b^ | <0.001 |
| Duration of Gn (d) | 10 (9, 12) | 10 (9, 11) | 9 (9, 10) | 10 (8, 11) | 0.098 |
| Total dose of Gn (IU) | 1950  (1500, 2550) | 2250  (2025, 2550) | 2250  (1500, 2850) | 2100  (1350, 2700) | 0.595 |
| E2 in hCG day (pg/mL) | 2820.24  (2282.32, 3222.14) | 2856.80  (2227.66, 4381.57) | 1367.44  (773.30, 1960.11) ^a,b^ | 1193.97  (589.43, 1603.05) ^a,b^ | <0.001 |
| Retrieved oocyte (n) | 11 (9, 14) | 11 (9, 16) | 4 (2, 5) ^a,b^ | 2 (1, 5) ^a,b^ | <0.001 |
| Fertilized oocyte (n) | 8 (6, 10) | 9 (5, 12) | 3 (2, 4) ^a,b^ | 2 (1, 4) ^a,b^ | <0.001 |
| Total embryos number (n) | 8 (6, 9) | 9 (6, 11) | 3 (2,4) ^a,b^ | 2 (1, 3) ^a,b^ | <0.001 |
| Good-quality embryo number (n) | 3 (2, 5) | 3 (1, 4) | 2 (0, 2) ^a,b^ | 0 (0, 1) ^a,b^ | <0.001 |
| Chemical pregnancy rate (%) | 57.1 (12/21) | 35.7 (5/14) | 50.0 (7/14) | 44.44 (5/9) | 0.641 |
| Clinical pregnancy rate (%) | 47.62 (10/21) | 35.7 (5/14) | 42.9 (6/14) | 33.33 (3/9) | 0.407 |

Values were presented as median (25^th^, 75^th^) or percentages (numbers).

^a^, significant different from Group A; ^b^, significant different from Group B; ^c^, significant different from Group C.
