# Supplementary material for: GPD1L‐Mediated Glycerophospholipid Metabolism Dysfunction in Women With Diminished Ovarian Reserve: Insights From Pseudotargeted Metabolomic Analysis of Follicular Fluid
Source: Cell Prolif. 2025 Mar 20;58(9):e70024. doi: 10.1111/cpr.70024 (PMC12414641; doi:10.1111/cpr.70024)
Supplement: Supplementary file 3 — Table S2. [file CPR-58-e70024-s002.docx]

**Table S2. Primer sequences for RT-qPCR**

| Gene name | Primer sequence |
| --- | --- |
| Human GPD1L | Forward: 5‘-ATCAAGGGCATAGACGAGGG -3’  Reverse: 5‘-TCTGCATCATCAACCACGGTA-3’ |
| Human ACTB | Forward: 5‘-ATCGTCCACCGCAAATGCTTCTA-3’  Reverse: 5‘-AGCCATGCCAATCTCATCTTGTT-3’ |
| Human NRF1 | Forward: 5‘-GCAGCCGCTCTGAGAACTT-3’  Reverse: 5‘-AGGCGAGTCTTCATCAGCAC-3’ |
| Human PGC1α | Forward: 5‘- TGAAGACGGATTGCCCTCATT -3’  Reverse: 5‘- GCTGGTGCCAGTAAGAGCTT -3’ |
| Human OPA1 | Forward: 5‘- GCCCTTCCTAGTTCAGAAGACC -3’  Reverse: 5‘- GCTCACCAAGCAGACCCTTT -3’ |
| Human MFN2 | Forward: 5‘- CTCTCGATGCAACTCTATCGTC -3’  Reverse: 5‘- TCCTGTACGTGTCTTCAAGGAA -3’ |
