# Supplementary material for: GPD1L‐Mediated Glycerophospholipid Metabolism Dysfunction in Women With Diminished Ovarian Reserve: Insights From Pseudotargeted Metabolomic Analysis of Follicular Fluid
Source: Cell Prolif. 2025 Mar 20;58(9):e70024. doi: 10.1111/cpr.70024 (PMC12414641; doi:10.1111/cpr.70024)
Supplement: Supplementary file 4 — Table S3. [file CPR-58-e70024-s004.docx]

**Table S3. Antibodies for immunofluorescence and Western blot**

| Antigen | Company | Cat No. | Species | IF | WB |
| --- | --- | --- | --- | --- | --- |
| GPD1L | Proteintech | 17263-1-AP | Rabbit |  | 1:1000 |
| GPD1L | Santa cruz | sc-517404 | Mouse | 1:100 | 1:500 |
| BAX | Abmart | T40051F | Rabbit |  | 1:1000 |
| BCL-2 | Abmart | T40056F | Rabbit | 1:200 | 1:1000 |
| TOM20 | Santa cruz | sc-17764 | Mouse | 1:100 |  |
| DDX4 | abcam | ab27591 | Mouse | 1:200 |  |
| Ki67 | abcam | ab15580 | Rabbit | 1:200 |  |
| γ-H2A.X | abcam | ab2893 | Rabbit | 1:200 |  |
| β-actin | Proteintech | 66009-1-Ig | Mouse |  | 1:5000 |
| Goat Anti-Mouse IgG H&L (HRP) | abcam | ab205719 | Goat |  | 1:10000 |
| Goat Anti-Rabbit IgG H&L (HRP) | abcam | ab205718 | Goat |  | 1:10000 |
| Donkey anti Rabbit 568 | Invitrogen | A10042 | Donkey | 1:100 |  |
| Donkey anti Mouse 488 | Invitrogen | A21202 | Donkey | 1:100 |  |
| Donkey anti Mouse 568 | Invitrogen | A10037 | Donkey | 1:100 |  |
