# Supplementary material for: GPD1L‐Mediated Glycerophospholipid Metabolism Dysfunction in Women With Diminished Ovarian Reserve: Insights From Pseudotargeted Metabolomic Analysis of Follicular Fluid
Source: Cell Prolif. 2025 Mar 20;58(9):e70024. doi: 10.1111/cpr.70024 (PMC12414641; doi:10.1111/cpr.70024)
Supplement: Supplementary file 5 — Data S1. Materials and Methods. [file CPR-58-e70024-s005.docx]

**Materials and methods**

**Study population**

The study included follicular fluid samples obtained from 60 women with diminished ovarian reserve (DOR group) and 60 healthy controls (normal ovarian reserve, NOR group) receiving *in vitro* fertilization/intracytoplasmic sperm injection and embryo transfer (IVF/ICSI-ET) procedures at the Department of Human Reproductive Medicine, Beijing Obstetrics and Gynecology Hospital. Women in the NOR group sought infertility treatment caused by tubal obstruction or male factors. The inclusion criteria for DOR patients were as follows^1^: (1) serum anti-Müllerian (AMH) level < 1.1 ng/mL, basal serum follicle-stimulating hormone (FSH) ≥ 10 IU/L or antral follicle counts (AFC) < 5-7; (2) regular menstrual cycles (28-35 days). Each group was further divided into two subgroups, including young and aged groups, based on an age of 35. Informed written consent was obtained from all the participants.

**Collection of follicular fluids**

The follicular fluids were collected at the time of oocyte retrieval from each patient during IVF/ICSI-ET procedures. Briefly, oocytes were retrieved by ultrasound-guided transvaginal follicular aspiration. The follicular fluids from mature follicles (diameter over 17 millimeters) were isolated and collected individually into a test tube and centrifuged at 3000 rpm for 10 minutes (min). The supernatant was further collected and stored at -80 °C for further analysis.

**Sample preparation**

The follicular samples were thawed on ice and vortexed for 30 s. 200 µL for each sample was transferred to an EP tube and 800 µL extraction solution (methanol: acetonitrile = 4:1, v/v) containing internal standards (IS) was added for protein precipitation. The samples were subsequently mixed on a vortex for 60 s and centrifuged at 15,000 *g* for 10 mins at 4 °C. The 900 μl of each sample was transferred to a centrifuge tube and lyophilized in a centrifugal vacuum evaporator at 4 °C. Next, reconstitute the samples in 60 μl of 90% H_2_O/CH_3_OH (vol/vol), vortex for 60 s and centrifuge for 10 mins at 15000 *g* for 10 mins. Transfer the supernatant to a threaded screw-neck vial for further analysis. Take 10 µl of the supernatant from the representative samples and mix them to prepare QCs to assess the stability of the analytical method.

**Pseudotargeted metabolomics detection process**

The specific analysis method for the pseudotargeted metabolomics referred to the existing protocol^2^. Briefly, the detection process can be divided into the following procedures: (1) untargeted metabolic proﬁling data were collected by using ultra-high-performance liquid chromatography–high-resolution mass spectrometry (UHPLC-HRMS) based on a Waters Acquity UHPLC system (Waters) coupled to an AB Sciex Triple Q-TOF 5600+ system (AB Sciex). Information-dependent acquisition (IDA) was used to obtain MS^2^ information. 5 µl of each reconstituted sample was injected onto a UHPLC-HRMS with IDA mode and six independent analyses were performed with different collision energy (CE) voltages (15, 30 and 45 V in positive ion mode and -15, -30 and -45 V in negative ion mode). (2) metabolic proﬁling data containing MS^2^ information was obtained and converted to XCMS-supported data type and mgf ﬁles using MSConvert. XCMS and CAMERA were used for peak detection and peak annotation and then multiple reaction monitoring (MRM) transitions were defined by MRM-Ion Pair Finder (version 2.0) written by R (version 3.6). (3) transformation of MRM transitions from HRMS to TQMS: inject 5-10 µl of IS reconstitution solution onto the UHPLC-TQMS system to obtain the retention time of each IS, and then the retention-time calibration was performed based on ISs with a ready-made function in R statistical scripting language to reduce the effect of different retention times between UHPLC-HRMS and UHPLC-TQMS. Three injections are performed near the recommended optimized CE value (CE of HRMS and ± 5 eV) to select the CE value with better response for UHPLC-TQMS analysis. (4) evaluation of the analytical characteristics of the pseudotargeted metabolomics method, including the linearity, repeatability and stability.

For large-scale metabolomics research, 10 QC samples were run for instrument balance at the start of each batch and a QC was inserted in every 5-15 injections for signal drift calibration. Extract peak areas using the software MultiQuant from AB Sciex. Metabolites that were detectable in < 80% of the samples in each sample group and metabolites with CV values >30% in QC samples after peak area standardization by ISs were removed. The pre-processed pseudotargeted metabolomics data were used for further statistical analysis.

**Data processing**

The data was unit variance scaled before unsupervised principal component analysis (PCA), which was performed by statistics function prcomp within R (www.r-project.org). The orthogonal partial least squares discriminant analysis (OPLS-DA) models were constructed using the R package MetaboAnalystR, before which the data was log transform (log_2_) and mean centering. The variable importance in the projection (VIP) values of metabolites in each model was calculated based on OPLS-DA results. Subsequently, the differential metabolites in each group were identified by VIP >1, *P* < 0.05 (Mann‒Whitney U test), and |fold change (FC)| > 1.2. Then, the Kyoto Encyclopedia of Genes and Genomes (KEGG) Compound database (http://www.kegg.jp/kegg/compound/) was performed to annotate the identified metabolites and then mapped to the KEGG Pathway database (http://www.kegg.jp/ kegg/pathway.html).

**Human granulosa cell collection**

Human granulosa cells (hGCs) were isolated from follicular fluids obtained during oocyte retrieval. In this, follicular fluid was centrifuged at 400 × *g* for 10 mins, and the layer of granulosa cells with red blood cell pellet was resuspended in Dulbecco’s modified Eagle medium/nutrient mixture F-12 Ham (DMEM/F-12, Gibco, USA). Then, 7.0 ml cell resuspension was layered on 8.0 mL Ficoll-Paque Plus (Cytiva, USA) and centrifuged at 600 × *g* for 20 min. Granulosa cells at the interface were subsequently harvested and washed with DMEM/F-12 three times and the cell pellets were stored at -80°C until the next analysis.

**Cell culture and transfection of small interfering RNA**

Human granulosa-like tumor cell line (KGN cells) utilized for the functional analysis in this study was purchased from Beijing Beina Chuanglian Biotechnology Institute. KGN cells were cultured in DMEM/F-12 medium supplemented with 10% fetal bovine serum (FBS, Gibco) and 1% penicillin-streptomycin (PS, Gibco) and incubated at 37°C in a humid atmosphere with 5% CO_2_. To simulate the chronic oxidative stress state in ovarian aging, 50 μM hydrogen peroxide (H_2_O_2_) was supplemented into the culture medium of KGN cells for 7 days.

To downregulate the expression of *GPD1L*, KGN cells were transfected with short interfering RNAs (siRNAs) purchased from Ribobio (China). KGN cells were seeded in 6-well plates, cultured overnight and transfected with siRNA when the cells reached 30%-50% confluent using INTERFERin (PolyPlus, France) according to the manufacturer’s protocol. RT-qPCR was performed to confirm the efficiency of transfection after incubation for 48 hours (h) and Western blotting (WB) was performed after 72 to 96 h. The siRNA sequences are as follows: si-GPD1L#1: 5’- GCAGACCAGTTCAAGAGAT-3’, si-GPD1L#2: 5’- GTTGCCATGTCAAATCTTA-3’.

**RT-qPCR**

Total RNA was extracted from cells using TRIzol reagent (Invitrogen) and then reverse-transcribed into complementary DNA (cDNA) using HiScript III All-in-one RT SuperMix Perfect for qPCR (Vazyme, China) according to the manufacturer’s instructions. For amplification, quantitative polymerase chain reaction (qPCR) was conducted with LightCycler480 real-time PCR system (Roche, Sweden) using TB Green® Premix Ex Taq^TM^ II (Takara, Japan). Samples were normalized to the general housekeeping gene *ACTB*, and the expression levels were calculated relative to the negative control groups using the 2^−ΔΔCt^ method. The primer details are provided in Table S2.

**Western blots**

Cell protein lysates were extracted using radioimmunoprecipitation assay (RIPA) lysis buffer containing protease inhibitor cocktail (EpiZyme, China) and phosphatase inhibitor (EpiZyme, China). Then, 25 μg protein was loaded into an 8-16% sodium dodecyl sulfate-polyacrylamide gel electrophoresis (SDS-PAGE) and electrotransferred onto PVDF membranes. After blocking with 5% skim milk in Tris-buffered saline solution with 0.1% Tween-20 (TBST) for 1 h, the membranes were incubated with primary antibodies (Table S3) overnight at 4°C and then were washed and incubated with corresponding horseradish peroxidase (HRP)-conjugated secondary antibodies (Table S3) for 1 h at room temperature (RT). The intensities were quantified via the enhanced chemiluminescence system (Piece, Rockford). The densitometric quantification was performed using ImageJ software (NIH) and the ratio to ACTB was calculated, given as fold changes and the values of negative control groups were set as 1.

**Cell sample preparation for quantitative lipidomics analysis**

KGN cells transfected with *GPD1L* siRNA for 4 days were used for quantitative lipidomics analysis. Cell samples were thawed on ice and 100 μl of ultrapure water extract (containing protease inhibitors, PMSF and EDTA) was added. 50 μl cell suspension was added with 500 μl mixture (methanol, MTBE and internal standard mixture). Vortex the mixture for 15 min, adding 100 μl of water and vortex for another 1 min, and then centrifuge it with 12,000 rpm at 4 ℃ for 10 min. Extract 300 μl supernatant and concentrate it. Dissolve powder with 200 uL reconstituted solution and take the dissolving solution into the sample bottle for LC-MS/MS analysis. The left 50 μl cell suspension was frozen and thawed 3 times, centrifuged at 12,000 rpm for 10 min. The supernatants were taken to determine the protein concentration by BCA Protein Assay kit.

**Quantitative lipidomics analysis**

The sample extracts were analyzed using an LC-ESI-MS/MS system (UPLC, ExionLC AD, https://sciex.com.cn/; MS, QTRAP® 6500+ System, <https://sciex.com/>). The effluent was alternatively connected to an ESI-triple quadrupole-linear ion trap (QTRAP)-MS. LIT and triple quadrupole (QQQ) scans were then acquired on a triple quadrupole-linear ion trap mass spectrometer (QTRAP), QTRAP^®^ 6500+ LC-MS/MS System, equipped with an ESI Turbo Ion-Spray interface, operating in positive and negative ion mode and controlled by Analyst 1.6.3 software (Sciex). Instrument tuning and mass calibration were performed with 10 and 100 μmol/L polypropylene glycol solutions in QQQ and LIT modes, respectively. QQQ scans were acquired as MRM experiments with collision gas (nitrogen) set to 5 psi. DP and CE for individual MRM transitions were done with further DP and CE optimization. A specific set of MRM transitions was monitored for each period according to the metabolites eluted within this period. Lipid contents were detected by MetWare (http://www.metware.cn/) based on the AB Sciex QTRAP 6500 LC-MS/MS platform. Data analyses were performed using the Metware Cloud, a free online platform (https://cloud.metware.cn).

**Cell Counting Kit-8 assay**

To detect the cell viability, the cell proliferation was determined by Cell Counting Kit-8 (CCK-8; Lablead, China) assay. Three days after the siRNA transfection, KGN cells were seeded into 96-well culture plates at a density of 2,000 cells/well. After incubation for 24, 48, and 72 h, 10 μl CCK-8 solution was added to each well respectively and incubated at 37°C for another 2 h. The PerkinElmer/Ensight (USA) was conducted to measure the absorbance at 450 nm.

**EdU assay**

KGN cells transfected with siRNA were incubated with 5-ethynyl-20-deoxyuridine (EdU, Ribobio, China) for 2 h, according to the manufacturer’s instructions. After washing with PBS 3 times, the cells were fixed in 4% PFA for 30 min and then incubated with 2 mg/ml glycine, after which the cells were permeabilized in PBS with 0.5% Triton X-100 for 10 min. Next, the cells were incubated in 1 × Apollo^®^ 567 reaction cocktail for 30 min and the cell nuclei were stained with 5 μg/ml Hoechst 33342 for 10 min. Images were captured under a confocal laser scanning microscope (LSM880, Carl Zeiss, Germany).

**TUNEL assay**

The apoptotic status of granulosa cells in *Gpd1l*-KD mouse ovaries were detected by TdT-mediated dUTP nick end labeling (TUNEL) Apoptosis Assay Kit (Solarbio, China) following the instructions. Briefly, the paraffin-embedded ovarian sections were deparafﬁnized, rehydrated with 100%, 95%, 85%, and 75% alcohol and washed in phosphate-buffered saline (PBS). Then, the slides were permeabilized in 20 μg/ml Proteinase K for 20 min at 37 °C. After being washed with PBS two times, the slides were incubated with TUNEL working solution for 2 h at 37 °C and then washed in 0.1% Triton X-100 containing 5 mg/ml bovine serum albumin (BSA, Sigma-Aldrich, USA). Then the cell nuclei were stained with DAPI for 10 min. The images were captured by laser scanning confocal microscope (Zeiss LSM880, Germany).

**Flow cytometry analysis**

Mitochondria mass was detected with an Acridine Orange 10-Nonyl Bromide (NAO) fluorescence probe (Solarbio, China). KGN cells were stained with 1μM NAO at 37°C in the dark for 20 min. For cell apoptosis, mitochondria membrane potential (MMP) and reactive oxygen species (ROS) level detections, 300 μM H_2_O_2_ was supplemented into the culture medium for 24 h 4 days after siRNA transfection. Cell apoptosis level was detected by Annexin V-FITC apoptosis Detection Kit (Beyotime, China). Cells were stained in 200 μl binding buffer with 5 μl Annexin V-FITC and 10 μl PI reagent in the dark for 20 min at RT. 2',7'-Dichlorodihydrofluorescein diacetate (DCFH-DA, Solarbio, China) was used to determine the ROS level, in which cells were incubated in 10 μM DCFH-DA diluted in Opti-MEM for 30 min at 37°C in the dark. JC-10 (Lablead, China) was utilized to examine the MMP, cells were resuspended in 1 μl JC-10 probe (200 ×) with 200 μl binding buffer in the dark for 20 min at 37°C. Flow cytometric analysis was performed with a BD FACS Aria Fusion (BD Biosciences, USA). Data analysis was performed using FlowJo^TM^ 10 software (TreeStar, USA).

**NADH/NAD^+^ assay**

The intracellular NADH/NAD^+^ levels were measured with the NAD^+^/NADH Assay Kit (WST-8, Beyotime, China) according to the manufacturer's instructions. Briefly, 1 × 10^6^/well cells were lysed with 200 μl pre-cooled NADH/NAD^+^ lysis buffer after removing the medium and centrifugated at 12,000 *g* for 10 min at 4°C. Then, 50-100 μl supernatant was moved to a tube, followed by incubation at 60°C for 30 min, and centrifugated at 10,000 *g* for 5 min at 4 °C. 20 μl supernatant or standard samples were transferred into 96-well plates and 90 μl alcohol dehydrogenase working fluid was added to each well, respectively. Next, after incubating at 37°C for 10 min, then 10 μl color-developing solution was added. The ratio of NADH/NAD^+^ was measured by detecting the absorbance at 450 nm using a microplate reader (Ensight, PerkinElmer, USA).

**ATP assay**

The intracellular ATP levels were measured with the ATP Assay Kit (Beyotime, China) according to the manufacturer’s instructions. Cells were lysed with lysis buffer and centrifuged at 12,000 × *g* for 5 min at 4°C. 20 µl supernatant was then added to 100 µl ATP detection working dilution in a 96-well plate. The luminescence of each sample was measured with a luminometer (Ensight, PerkinElmer, USA) and the protein concentration was detected using the BCA Protein Assay Kit (Beyotime, China). The relative ATP level was calculated as ATP value/protein value.

**Immunofluorescence staining**

The paraffin-embedded ovarian sections were deparafﬁnized, rehydrated and washed in PBS. The slides were boiled in Tris-EDTA buffer for 15 min for antigen retrieval and cooled to RT. Then, the slides were permeabilized in 0.5% Triton X-100 for 10 min, followed by blocking with 3% BSA. Next, the sections were incubated with primary antibodies (Table S3) at 4 °C overnight. After being washed with PBS, the slides were incubated with corresponding secondary antibodies (Table S3) for 1 h at RT, subsequently with DAPI for 10 min. Fluorescent images were observed and captured with a laser scanning confocal microscope (LSM880, Carl Zeiss, Germany). The same fluorescence channels were acquired by the same immunostaining procedures and threshold value.

KGN cells transfected with *GPD1L* siRNA were fixed in 4% paraformaldehyde (PFA) for 30min and permeabilized in 0.25% Triton X-100 for 10 min, followed by blocking in 2% BSA for 60 min. Then, the cells were incubated with primary antibodies at 4°C overnight and incubated with corresponding secondary antibodies (Table S3) for 1 h at RT. The cell nuclei were stained with DAPI for 10 min. Images were captured using a laser-scanning confocal microscope (Leica Stellaris, Germany). Image analyses were performed using ImageJ software (NIH).

**Animals and *in situ* microinjection in mouse ovaries**

Three-week-old ICR mice were purchased from the SPF Biotechnology Co., Ltd. (China) and housed in the Animal Laboratory Center, Institute of Zoology, Chinese Academy of Sciences under 12/12 h light/dark cycles with food and water available ad libitum. The experiments involving mice were approved by the Animal Research Committee and the Ethics Committee of Beijing Obstetrics and Gynecology Hospital, Capital Medical University. For adeno-associated virus (AAV) microinjection, the mice were placed in a prone position after anesthesia and then the back skin and muscles were incised. 6 μl of Mouse_Gpd1l-shRNA-GPAAV-Egfp or Scramble-eGFP-AAV2/9 particles constructed by Genomeditech Co., Ltd. (China) were injected into the space between the membrane and ovary with 34G needle in viewing under the microscope. One week after the AAV infection, mice were sacrificed, the ovaries were collected and stored at -80°C for further analysis.

**Estrous cycle determination**

The estrus cycle stages were determined based on vaginal exfoliated cell cytology under microscopic analysis. Briefly, mice vaginal exfoliated cells were collected daily at 9:00 AM for 15 consecutive days one week after the AAV microinjection. A pipette gun was used to inject 10 μl of the saline solution into the vagina, and the rinse fluid was then aspirated, transferred onto glass slides. Then, the dried smears were stained with Wright’s Stain solution (Solarbio, China) for 3 min, mixed with an equal amount of distilled water, and stained for another 5 min. The stages of the estrus cycle were determined as follows: proestrus stage (nucleated epithelial cells mainly), estrus stage (cornified epithelial cells mainly), metestrus (cornified epithelial cells and leukocytes), diestrus (mostly leukocytes).

**H&E staining and follicle counting**

The mice ovaries were fixed in 4% PFA for 6 h and dehydrated in graded alcohol (70%, 80%, 90%, and 100%). Ovarian tissues were then embedded in paraffin and cut into 5 μm serial sections with a rotary microtome (Leica, Germany). For H&E staining, the ovarian sections were deparaffinized with xylene and rehydrated with graded alcohol (100%, 95%, 85%, and 75%), followed by incubating in hematoxylin solution for 4 min. The sections were then washed with running tap water for 10 min and incubated in 1% acid alcohol for 5 s. After washing with water for 3 min, the slides were incubated in eosin for 8 min and dehydrated in graded ethanol series (95%, 100%, and 100%), immersed with xylene. Finally, the slides were sealed with coverslips. Images were captured using Leica Aperio VESA8 (Germany).

The number of follicles at different developmental stages (primordial, primary, secondary, antral, preovulatory and atretic follicles) was counted blindly in every fifth section throughout the ovary according to the morphological criteria^3^. Follicles with distinct oocyte nuclei were counted to avoid overcounting. The total number of resting follicles (primordial and primary follicles) required to be multiplied by a correction factor of five.

**Superovulation**

To collect oocytes for quality assessment, mice were intraperitoneally injected with pregnant mare serum gonadotropin (PMSG, Sansheng Pharmaceutical, China) one week after the AAV microinjection. After 48 h, human chorionic gonadotrophin (hCG) was administered via intraperitoneal injection and 14-15 h after the hCG injection, the mice were sacrificed and the oviducts were collected. COCs were then released by puncturing the oviducts under a microscope and enzymatically dissociated with 10 mg/ml hyaluronidase (Millipore-Sigma, Germany). The collected oocytes were maintained separately in 0.1% BSA for further analysis.

**Oocyte quality assessment**

Annexin V-mCherry Apoptosis Detection Kit (Beyotime, China) was conducted to examine the apoptosis level of oocytes. Oocytes were incubated in 195 μl binding buffer supplemented with 5 μl Annexin V-mCherry for 20 min at RT in the dark. MitoTracker® Red CMXRos (Solarbio, China) was used to test the mitochondrial activity, in which oocytes were incubated in 200 nM mitotracker with OptiMEM medium (Gibco, USA) for 30 min at 37°C. JC-10 and DCFH-DA were applied to detect oocyte MMP and ROS levels, respectively, and the experimental methods were as described above. Finally, oocytes were washed with PBS containing 0.1% BSA three times, transferred, and covered with a thin layer of mineral oil. The images were captured using a fluorescence microscope (LSM880 Fast Ariyscan, Carl Zeiss, Germany). The intensities analysis was performed using ImageJ software (NIH).

**Statistical analysis**

Each experiment was performed at least three times independently. All data were reported as mean ± standard error of the mean (SEM). Unpaired Student’s two-tailed *t*-test or Mann-Whitney U test was utilized for parametric tests and nonparametric tests respectively between the two groups, and comparisons of multiple groups were analyzed by one-way ANOVA. The correlation between the variables was performed using Pearson rank correlation analysis. Statistical analyses were performed with GraphPad Prism 10 (GraphPad Software, USA). *P*-value < 0.05 was regarded as statistically signiﬁcant. Statistical significance was defined as **P* < 0.05, ***P* < 0.01, ****P* < 0.001 and *****P* < 0.0001, respectively.

**References**

1. Pastore LM, Christianson MS, Stelling J, Kearns WG, Segars JH. Reproductive ovarian testing and the alphabet soup of diagnoses: DOR, POI, POF, POR, and FOR. *J Assist Reprod Genet.* 2018;35(1):17-23.

2. Zheng F, Zhao X, Zeng Z, et al. Development of a plasma pseudotargeted metabolomics method based on ultra-high-performance liquid chromatography-mass spectrometry. *Nat Protoc.* 2020;15(8):2519-2537.

3. Myers M, Britt KL, Wreford NG, Ebling FJ, Kerr JB. Methods for quantifying follicular numbers within the mouse ovary. *Reproduction.* 2004;127(5):569-580.
